# Damage signals preferentially activate killer CD8^+/−^ regulatory T cells to protect injured tissue

**DOI:** 10.1101/2025.01.13.632166

**Published:** 2025-01-14

**Authors:** Aditya Josyula, Daphna Fertil, Devon R. Hartigan, Paige Rudy, Sonakshi Sharma, Toluwaleke Fashina, Efua Maclean, Alessandra B. Coogan, Tran B. Ngo, Vanathi Sundaresan, Kaitlyn Sadtler

**Affiliations:** 1 Section for Immuno-Engineering, National Institute of Biomedical Imaging and Bioengineering, National Institutes of Health, Bethesda, MD 20892, USA; 2 Kavli Institute for Nanoscience Discovery, University of Oxford, Oxford, Ox13QU, UK; 3 Lead contact

## Abstract

Self-antigens that are obscure to immune cells under homeostasis, are exposed following tissue damage and can trigger autoimmunity. Our previous work showed that conventional type 1 dendritic cells (cDC1s) mediate immunoregulation after traumatic skeletal muscle injury. Here, we found that, immune responses to injury in cDC1-depleted mice (*Batf3*^−/−^) mirror those of autoimmune mice (*Aire*^−/−^). Mechanistically, we determined that cDC1s prime killer regulatory T cells (CD8^+/−^Ly49^+^Tregs) which express Ly49 inhibitory receptors and HELIOS. These killer-like Tregs are clonally diverse and carry T cell receptors associated with self-reactivity and response to hydrophobicity. cDC1 or CD8 deletion promoted CD62L^+^CCR7^+^ naïve T cell retention and B cell recruitment to injured muscle. These naïve T cells, which are implicated in autoimmunity, strongly correlate with B cell abundance in the muscle and are selectively pruned by CD8^+/−^Ly49^+^ Tregs. Furthermore, clinically used materials that promote wound healing enrich CD8^+/−^Ly49^+^ Treg function whereas those that are associated with pathology promote naïve T cell and B cell accumulation. We hypothesize that CD8^+/−^Ly49^+^ Tregs maintain self-tolerance after tissue damage and avert autoimmunity by eliminating naive T cells and preventing pathogenic B cell activation.

## INTRODUCTION

A major feature of the immune system is the ability to recognize danger and pathogen from homeostasis and self-antigen^1,2^. Upon tissue damage, self-antigens that are otherwise obscured from immune cells become exposed. T cells that bind self-antigens are centrally deleted in the thymus or converted into regulatory T cells, and secondary mechanisms of protection such as peripheral tolerance induction prevent self-reactive T cells from escaping tolerance. However, chronic inflammation in non-lymphoid tissues can subvert these mechanisms and give rise to autoimmune pathologies and fibrotic scarring^3^.

Induction of autoimmune diseases is sometimes associated with a trigger event such as infection or surgery, suggesting that self-antigen exposure following tissue damage drives pathology^4–6^; however, a large majority of these patients do not develop autoimmunity owing to multiple mechanisms of T cell regulation. Certain immunologic phenotypes are well documented in autoimmune pathologies that follow tissue damage in humans, such as autoantibody generation, increased activation of CD8^+^ T cells, and over-expression of inflammatory cytokines^7^. Furthermore, in response to allogeneic transplants and chronic inflammation after infection, naïve T cells accumulate in non-lymphoid tissues, organize into tertiary lymphoid structures and can generate an ectopic germinal center response potentiating autoimmunity^8^. In contrast to these pathologies, in murine models of healthy tissue repair, IL-4 producing Th2 T cells have been found to promote myoblast fusion in the muscle, amphiregulin-producing CD4^+^FoxP3^+^ regulatory T cells which promote tissue repair through macrophage repolarization, and MAIT cells promote epithelial regrowth in the skin^9–12^. Therefore, preventing autoimmunity is a prerequisite for healthy tissue repair. Given the large number of patients with traumatic injuries and surgically induced tissue damage, and autoimmune-connected syndromes, a way to target and promote immunologic self-tolerance after tissue damage is needed for furtherance of patient care.

Previously, using a volumetric muscle loss (VML) model in mice, we identified conventional dendritic cell type 1 (cDC1) which was recruited to injured skeletal muscle through XCL1 chemotaxis and was associated with T cell and macrophage immunoregulation with downstream effects on stem cell differentiation and tissue development^13^. These cells were enriched by a clinically used therapeutic biomaterial, decellularized extracellular matrix (ECM), associated with improved outcomes in wound healing and were inhibited by the presence of hydrophobic plastic wear particles (polyethylene, PE) as seen in implant pathologies^14,15^. Here, using high dimensional gene and protein-level analyses of dendritic cells and lymphocytes responding to VML, we investigated cDC1-T cell communication as a potential mechanism of self-tolerance after traumatic injury. In muscles with a pro-regenerative environment induced by implanting ECM at the injury site we found that a CD4 negative Ly49 inhibitory receptor positive (Ly49^+^) and HELIOS^+^ regulatory T cell subset (CD8^+/−^Ly49^+^ Tregs) was abundant. cDC1s communicate with Ly49^+^ Tregs and stimulate their proliferation when loaded with ECM proteins in vitro. In vivo, we observed that downstream of cDC1 stimulation, CD8^+/−^Ly49^+^ Tregs impede naïve T cell accumulation in non-lymphoid tissue, which is known to instigate tertiary lymphoid structure formation, decreasing local B cell accumulation and averting autoimmunity.

## RESULTS

### Mice that lack cDC1s mirror the phenotype of mice that lack self-tolerance with heightened inflammation in mice with hydrophobic plastic implants

**Figure 1 |:**
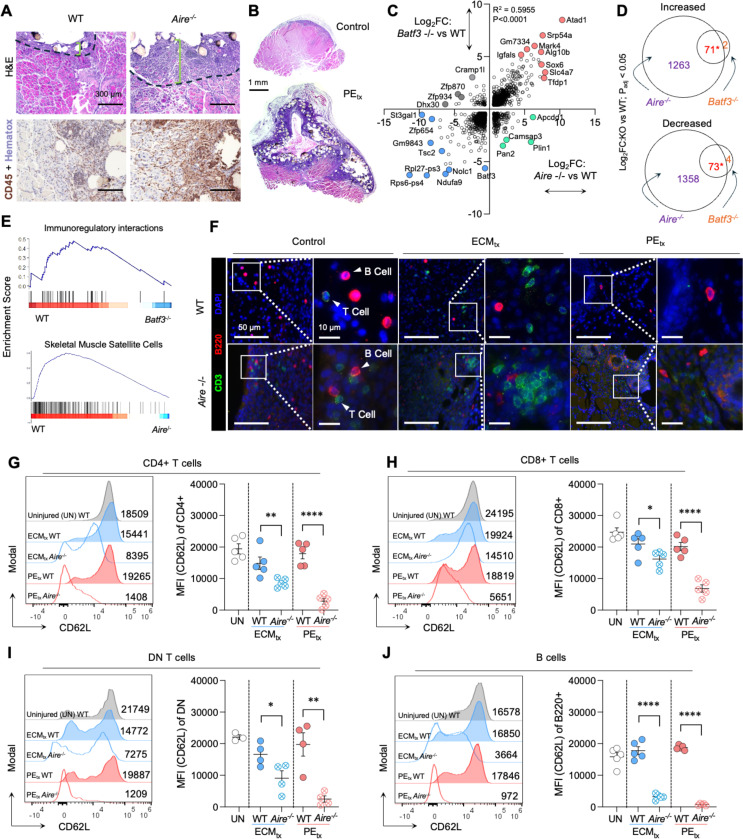
Mice that lack cDC1s mirror and autoimmune phenotype with heightened inflammation in muscle injury when exposed to the plastic implant polyethylene. (**A**) Hematoxylin and eosin (H&E) and CD45 staining of WT and *Aire*^−/−^ mice at 7 days post-injury (dpi) with PE_tx_. (**B**) Whole cross-section of control and PE_tx_ injury in *Aire*^−/−^ mice. (**C**) Bulk RNAseq of *Batf3*^−/−^ and *Aire*^−/−^ muscle tissue at 7 dpi. (D) Shared gene differences in *Aire*^−/−^ and *Batf3*^−/−^ mice. (**E**) Gene set enrichment analysis of bulk RNAseq. (**F**) Immunofluorescence microscopy of muscle tissue at 7dpi in WT vs *Aire*^−/−^ mice, red = B220, green = CD3, blue = DAPI. (**G-J**) CD62L expression in WT and *Aire*^−/−^ mice on (**G**) CD4+ T cells (**H**) CD8+ T cells (**I**) Double negative (DN) T cells, and (**J**) B Cells in the inguinal lymph node of injured mice (7dpi). Data are mean ± standard error (SEM). Data in G-J are mean ± SEM in n≥3 mice. Data in C&D are n=3.

To model the base response of self-tolerance in traumatic skeletal muscle injuries under clinically relevant settings we employed the VML model with ECM (pro-healing biologic) or PE (pro-inflammatory and fibrotic plastic) biomaterial treatments. Histological examination of muscle tissue at 7 days post-injury (dpi) from *Aire*^−/−^ and wild type (WT) mice qualitatively showed increased infiltration of immune cells at injury site in *Aire*^−/−^ mice (Figure 1A). This immune response was highly amplified when hydrophobic particulate PE was implanted at the injury site (Figure 1B). As previously reported, tolerogenic cDC1s peak at 7dpi in the muscle and are critical for effective tissue repair^13^. To understand the contribution of tolerogenic cDC1s in restraining the autoimmune response, we performed bulk RNA sequencing of injured muscle tissue from *Aire*^−/−^, *Batf3*^−/−^ (cDC1-deficient) and WT mice at 7dpi (Figure 1C-D). Comparing differentially expressed genes in *Aire*^−/−^ and *Batf3*^−/−^ to WT controls revealed a highly overlapping expression pattern including upregulated *Atad1*, *Srp54a*, *Mark4* and *Slc4a7* and downregulated *Tsc2*, *Ndufa9*, and *Nolc1* (Figure 1C and Figure S1A). Broadly, tissue level differential expression of genes in *Batf3*^/-^ vs WT were nearly fully recapitulated within *Aire*^−/−^ phenotype (71 of 73 genes increased, 73 of 77 genes decreased, Figure 1D). Via gene set enrichment analysis (GSEA) we found that *Batf3*^/-^ mice were associated with decreased immunoregulatory interactions suggesting functional similarity to autoimmune-like responses and decreases in skeletal muscle satellite cells in *Aire*^−/−^ mice suggesting defects in regeneration as seen in histology (Figure 1E and Figure S1C-D).

Given these similarities between the cDC1-deficient and autoimmune mice, we investigated the difference in lymphocyte infiltration in *Aire*^−/−^ mice as we previously showed that cDC1-deficient mice had increased T cell activation in draining lymph nodes^13^. Compared to WT mice, CD3+ T and B220+ B cell infiltration of muscle tissue was amplified in *Aire*^−/−^ mice (Figure 1F and Figure S1B). Flow cytometric analysis of draining inguinal lymph nodes (ILN) showed significantly increased T and B lymphocyte activation determined by CD62L shedding in *Aire*^−/−^ mice as compared to WT mice (CD4+ T cells: ~1.8x p=0.0087 in ECM_tx_ and ~16x p<0.0001 in PE_tx_. CD8+ T cells: ~1.4x p=0.02 in ECM_tx_ and ~3.3x p<0.0001 in PE_tx_. DN T cells: ~2x p=0.02 in ECM_tx_ and ~16.4x p<0.0001 in PE_tx_. B cells: ~4.6x p<0.0001 in ECM_tx_ and ~18.4x p<0.0001 in PE_tx_ ; Figure 1G-J) consistent with previous findings in *Batf3*^−/−^ mice^13^. This phenotype was exacerbated with PE_tx_ across all lymphocytes evaluated including CD4+ T cells (Figure 1G), CD8+ T cells (Figure 1H), CD4-CD8- Double Negative (DN) T cells (Figure 1I) and B cells (Figure 1J). The large differences in lymphocyte activation were not explained by antigen experience as CD44 expression in *Aire* depleted mice was lower compared to WT mice (p<0.0001 in CD4+, CD8+, DN, and B cells in PE_tx_ ; Figure S1F). Furthermore, HELIOS expression in CD8+ but not CD4+ T cells in *Aire*^−/−^ mice which received either ECM_tx_ or PE_tx_ was significantly lower than WT mice which received control injury in peripheral blood (p<0.001 in ECM_tx_ and p=0.03 in PE_tx_) and ILN (p<0.0001 for both ECM_tx_ and PE_tx_, Figure S1E). This suggests that *Aire* deletion exacerbates muscle infiltration and draining lymph node activation of T and B cells and cDC1s are important regulators of this response.

### Material dependent recruitment of a diverse set of dendritic cells to skeletal muscle injury including cDC1s and pDC-like cells and CD310b+ DCs

**Figure 2 |:**
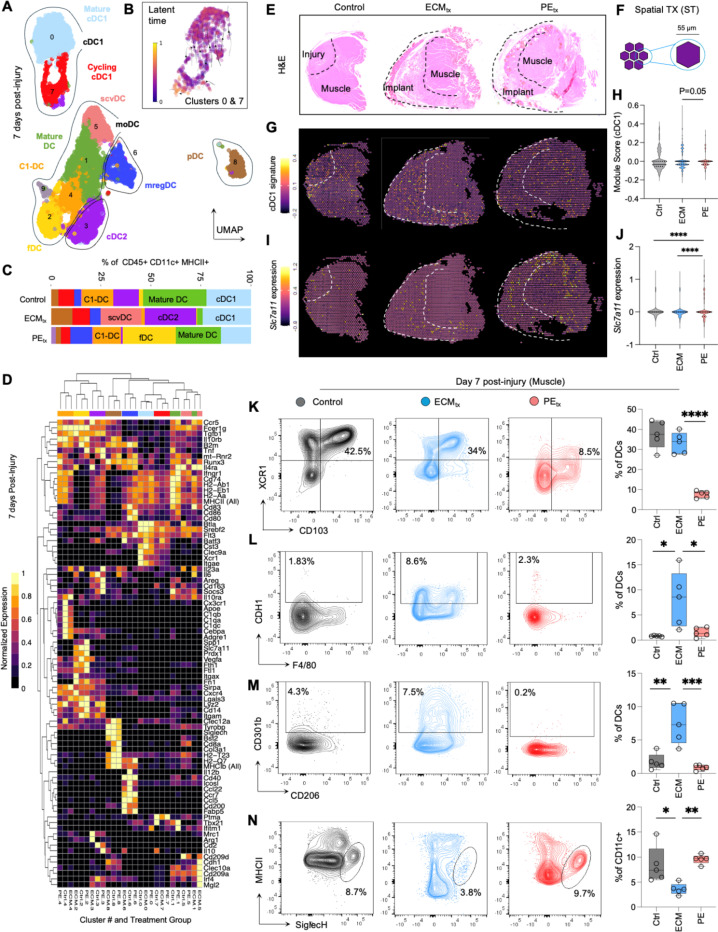
Material dependent recruitment of a diverse set of dendritic cells to skeletal muscle injury including cDC1s and pDC-like cells and CD301b+ DCs. (**A-B**) Uniform manifold approximation projection (UMAP) of (**A**) Seurat clusters and (**B**) Latent time analysis in CD11b^lo^MHCII^+^CD11c^+^ cells sorted from muscle at 7dpi, n=5 mice pooled. (**C**) Cluster prevalence across treatment groups. (**D**) Cluster phenotyping of key genes across treatment groups as normalized gene expression. (**E**) H&E of sections used for spatial transcriptomic analysis (**F**) Size scale of spatial sequencing data. (**G**) cDC1 gene signature (H) Module score of cDC1 signature. (**I**) *Slc7a11* gene expression and (**J**) Quantification of *Slc7a11* expression. (**K-N**) Dendritic cell phenotyping at 7dpi via flow cytometry of muscle detecting (**K**) cDC1s, (**L**) CDH1+ DCs, (**M**) scvDCs/CD301b+ DCs, (**N**) pDCs, n=5. Data are mean and distribution (**H,J**) or mean and interquartile range (IQR, **K-N**). Data in A-C are pooled n=5 mice. Data in E-J are n=1 mouse. Data in K-N represent mean ± SEM in n=5 mice.

Previously, we reported that regenerative materials such as decellularized ECM preferentially recruit cDC1s to the site of injury and material implantation and are associated with muscle regeneration. While cDC1s formed a large fraction of total DCs 1-week post-muscle trauma, heterogeneity within DC infiltrates was unknown. To probe DC diversity after trauma and material implantation, we isolated DCs (defined as CD45^+^SiglecF^-^F4/80^-^CD11c^+^MHCII^hi^) from mouse quadriceps 1-week post-injury and implantation including both biomaterial treatments (ECM and PE) and saline-treated injury control (Figure 2A-D). We analyzed a combined total of 9,377 DCs via single cell RNA sequencing (scRNAseq) that met filtering and quality control requirements. We identified 10 transcriptionally distinct, Seurat clusters confirmed by Uniform manifold approximation and projection (UMAP, Figure 2A and Figure S2A-B). We assigned cluster identity by analyzing differentially expressed transcripts from each cluster as compared to all other clusters and subsequently identifying characteristic transcripts evidenced in literature. cDC1s, type 2 classical dendritic cells (cDC2s), plasmacytoid-like DCs (pDCs), mature regulatory DCs (mregDCs), and several classes of monocytic DCs (moDCs) were detected. Through latent time analysis, we confirmed the transcriptional transition between proliferating (cluster 7, red) and mature (cluster 0, light blue) cDC1s (Figure 2B). Trauma alone in control mice resulted in recruitment of cDC1s (*Itgae, Xcr1*) and pDCs (*Siglech*) to the muscle (Figure 2C). Further, DCs enriched in *Klrd1, Clec10a, Cd209a* (Mature DCs) formed the largest subset in the control group. DC subsets in the control group were either expanded or depleted in a material dependent manner. Notably, cDC1s, *Cdh1* (which encodes for E-cadherin) and *Mgl2* (which encodes for CD301b) expressing DCs were enriched by ECM_tx_ and attenuated by PE_tx_. In contrast, DC clusters with *Ccr7* and *Slc7a11* were overrepresented in PE_tx._ While similar patterns were observed overall, there were variations across treatment groups within clusters of key genes used to identify dendritic cell subtypes (Figure 2D).

To assess spatial distribution of genes relevant to DC transcriptomes in the muscle, we harvested muscles at 7dpi, sectioned and stained with hematoxylin and eosin (Figure 2E) and performed spatial transcriptomics (ST, Figure 2F-J and Figure S2C). To visualize spatial distribution of key cell types, we utilized our single cell transcriptomics dataset as a reference and performed cluster-wise differential gene expression between subsets. We then created signature scores for cell types of interest and mapped the scores onto ST spots (Table S1). This approach revealed that cells expressing markers such as *Mgl2*, *Xcr1,* and *Slc7a11* were largely confined to regions with underlying tissue injury and material implants (Figure S2C). In contrast, *Ccr7* expression which was elevated in mreg DCs was more diffuse across tissue. Spatially, we saw an increased prevalence of the cDC1 signature in ECM_tx_ wounds (p=0.05 ECM_tx_ vs PE_tx_; Figure 2G-H) and an increased prevalence of *Slc7a11* fibrotic DCs (fDCs) in PE_tx_ wounds (p<0.0001 for control vs PE_tx_ and ECM_tx_ vs PE_tx_; Figure 2I-J).

Given the necessary sorting to enrich dendritic cells for sequencing and the differences between regulatory steps between gene and protein, we tested the validity of our findings at protein level via flow cytometry (Figure 2K-N and Figure S2D). We confirmed differential recruitment of cDC1s as seen in prior studies and sequencing analyses (p<0.0001 ECM_tx_ vs PE_tx_ ;Figure 2K). The enrichment of scavenger receptor positive cells expressing both CD301b (p=0.01 ECM_tx_ vs control and p=0.02 ECM_tx_ vs PE_tx_) and CDH1 (E-Cadherin) (p=0.0012 ECM_tx_ vs control and p=0.0004 ECM_tx_ vs PE_tx_) were detected in ECM_tx_ mice (Figure 2L-M). SiglecH^+^ “pDC-like” cells were represented in the CD11c^+^MHCII^lo^ population. These cells were elevated in controls (p= 0.01 vs ECM_tx_) and mice PE_tx_ (p=0.0039 vs PE_tx_) and lower in ECM_tx_ muscle (Figure 2N). Given the low levels of MHCII expression in pDCs this may explain the differences between cell prevalence in the sequencing and flow cytometry, highlighting the need to perform protein-level analyses on immune cells on intact immune populations.

### Recruitment of CD8^+/−^Ly49^+^ killer like regulatory T cells is enriched by ECM treatment and conserved across tissues

**Figure 3 |:**
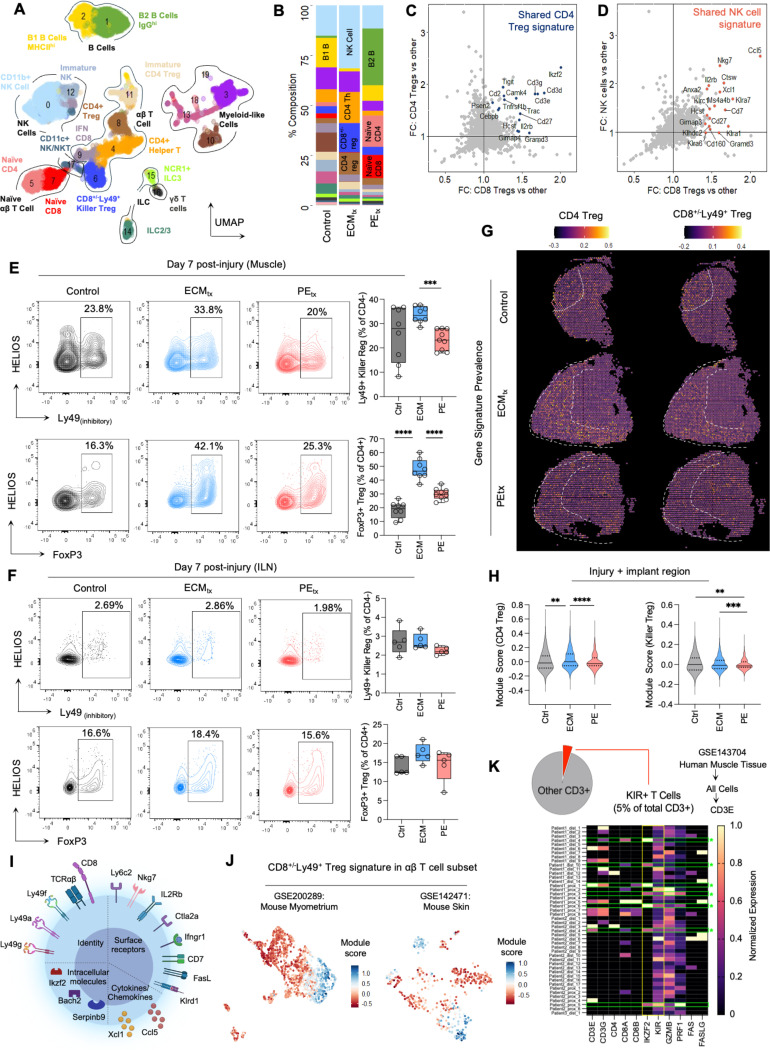
Recruitment of CD4^-^CD8^+/−^Ly49^+^ Killer regulatory T cells to the injury site is enriched by ECM_tx_ and is conserved across tissues. (**A**) UMAP of Seurat clusters of CD11b^lo^CD11c^lo^ cells sorted from muscle at 7dpi (**B**) Prevalence of clusters across treatment groups. (C-D) Shared gene signature of killer regulatory cells (cluster 6) with (**C**) Canonical CD4+ T regs (cluster 8) and (**D**) Natural Killer (NK) cells (cluster 0). (**E - F**) Flow cytometry analysis of canonical CD4 Tregs and CD8^+/−^Ly49^+^ Tregs in (**E**) muscle and (**F**) draining inguinal lymph node at 7dpi, n=5, data are mean ± IQR. (**G**) Spatial transcriptomic evaluation of CD4 and Killer Treg gene signatures with (**H**) module score. (**I**) Gene signature and profile of CD8^+/−^Ly49^+^ Tregs, Created in BioRender. (**J**) Mined single cell datasets for Killer Treg module score in mouse myometrium (GSE200289) and mouse skin (GSE132471). (**K**) Presence of CD8^+/−^Ly49^+^ Treg orthologs in human skeletal muscle detected via mined GSE143704 dataset. Data in A-D are a pooled n=10 mice. Data in E&F are mean ± SEM in n=5 mice. Data in G-H represent n=1 mouse.

T lymphocytes in the muscle, specifically IL-4 producing Th2 cells which are enhanced by ECM treatment, drive tissue regeneration in the muscle after trauma^10^. In addition, CD4^+^FoxP3^+^ regulatory T cells are preferentially recruited by ECM. Juxtaposing these phenomena, in up to 10% of patients who receive synthetic implants, symptoms of various autoimmune conditions arise transiently, receding after explantation. These reports led us to investigate specific lymphocyte subsets that drive autoimmune pathology and regulatory T cell subsets that restrain pathologic T cells to prevent tissue damage (Figure 3). To identify these subsets, we isolated all CD45+CD11b-CD11c- cells from muscle tissue at 7dpi and material implantation and performed scRNAseq. Multidimensional clustering of 13,334 cells revealed 15 non-myeloid subsets (Figure 3A-B). We identified 4 transcriptionally distinct subsets (CD4^+^Treg, CD4^-^Ly49^+^ Treg, NK Cell, and CD4^+^ Helper T) that were enriched by ECM_tx_ and lacking in PE_tx_, which implied an association with regenerative outcomes and 3 subsets (B2 B cell, Naïve CD4^+^, and Naïve CD8^+^) that were largely depleted by ECM_tx_ and enriched by PE which was suggestive of pathogenicity. Among the ECM_tx_ enriched T cell subsets were canonical *Cd4+Foxp3* expressing Tregs and CD4 T cells of Th2 phenotype. Curiously, we also observed a population of cells with low *Cd8a* expression and high *Ikzf2* and *Klra4/6/7* expression which has previously been defined as CD8 Tregs^16–20^. These cells represented a major fraction (~25%) of all sequenced αβT cells comparable to canonical *Foxp3* expressing CD4 Tregs (Figure 3B). Furthermore, by creating fold-change plots between *Cd4* and *Cd8a* expressing Tregs, we noted a shared phenotype between these subsets comprising *Il2rb, Cd27, Tigit*, and *Ikzf2* (Figure 3C). A similar analysis substituting CD4 T cells for NK cells showed a shared expression pattern comprising, *Ccl5, Nkg7, Xcl1* and *Klra7* (Figure 3D). Notably, both CD4 Tregs and NK cells were enriched within ECM_tx_. Sub-clustering the *Ikzf2* expressing *Cd4*^-^ T cells, we observed 3 distinct populations. Broadly, cells that expressed NKT cell markers, CD8+HELIOS+ Treg markers as well as markers previously ascribed to MAIT cells were enriched in the parent cluster. However, in the resulting sub-clusters, these defining markers were not defining features. This suggests that there are shared transcriptional programs which implied shared function, across all three Ly49+ T cell populations (Figure S3A-B). In contrast, PE_tx_ enriched both CD4 and CD8 T cells that expressed the gene encoding the homing receptor *Ccr7* and the naïve T cell marker *Sell* which encodes for CD62L.

Flow cytometric analysis of the muscle confirmed the elevated recruitment of regulatory T cells of both CD8 and CD4 lineage in ECM_tx_ to the muscle (p=0.019 for ECM_tx_ vs PE_tx_ in CD4-Ly49+ Tregs and p<0.0001 for control vs ECM_tx_ and ECM_tx_ vs PE_tx_ in CD4+FoxP3+ Tregs; Figure 3E and Figure S4A). Similar trends were observed in the draining inguinal lymph nodes (ILN), although not significantly different between treatment groups (Figure 3F). As previously reported, ECM_tx_ significantly enriched CD4+ T cells and PE_tx_ significantly enriched CD8+ T cells in the muscle at 7dpi (Figure S3C). Additionally, over a 6-week period, accumulation of neither CD62L-CD8+, CD62L-CD4+ nor CD62L- DN T cells in the muscle was treatment group dependent (Figure S3D). CD62L shedding on B cells was significantly greater in both PE_tx_ (p=0.032) and control (p=0.043) treatment groups as compared to ECM_tx_ suggesting B cell restraint in the muscle within ECM_tx_. Similarly, in agreement with our single cell transcriptomic dataset, naïve T cells, marked by lack of CD69 expression were elevated in PE_tx_ (p=0.0032 vs ECM_tx_) and control treatment (p=0.010 vs ECM_tx_) groups in the muscle (Figure S4B) but not the ILN (Figure S4C).

Next, we identified characteristic transcriptomic markers of CD4 and CD8 Tregs in the muscle and generated module scores using our single cell transcriptomics dataset (Table S1), then mapped the module scores onto spatial transcriptomics spots on muscle tissue across treatment groups (Figure 3G-H). In this analysis, both CD4 and CD8 Tregs were largely spatially confined to the injury and implantation sites at 7dpi suggesting that their effector functions are carried out in injured tissue (Figure 3G and Figure S3E&F). Further, the module scores were significantly elevated in control (p=0.0018; killer Treg) and ECM_tx_ (p=0.0009; killer Treg) as compared to PE_tx_ corroborating our observations using flow cytometry (Figure 3H). Indeed, broadly defining markers of lymphocyte subsets were spatially confined to the injury space with the exception of *Cd19* and *Ccr7* in PE_tx_ which were more widespread (Figure S3G-H). Since our animal model was based on sterile skeletal muscle injury, we next investigated whether this CD8^+/−^Ly49^+^ Treg signature is observed in other instances of tissue damage (Figure 3I). We mined publicly available single cell transcriptomic datasets of mouse skin^21^ and myometrium damage^22^ and looked for the CD8^+/−^Ly49^+^ Treg phenotype observed in the muscle in our animal model (Figure 3J). Indeed, CD8^+/−^Ly49^+^ Tregs represented ~ 10% of αβT cells in the context of skin injury and ~25% in the context of inflammation induced damage of mouse myometrium. Taken together, these findings suggest that CD8^+/−^Ly49^+^ Treg response is elicited upon tissue damage and is conserved across tissues. Given the differential immune responses between inbred laboratory mice and humans, we searched for this transcriptomic profile in publicly available datasets of human muscle tissue^23^. Using human orthologues of genes characteristic of CD8^+/−^Ly49^+^ Tregs in mice, we observed a similar CD8^+/−^Ly49^+^ Treg phenotype in the context of human muscle tissue damage (KIR^+^HELIOS^+^ T cells) thus extending the relevance to human tissue injury (Figure 3K). Previous reports of regulatory CD8+ T cell abundance in human airways after severe COVID-19 infection lends further credence to our damage response hypothesis^24^.

### CD8^+/−^Ly49^+^ killer regulatory cells have unique CDR3 repertoire and are not clonally expanded relative to canonical Tregs and helper T cells

**Figure 4.**
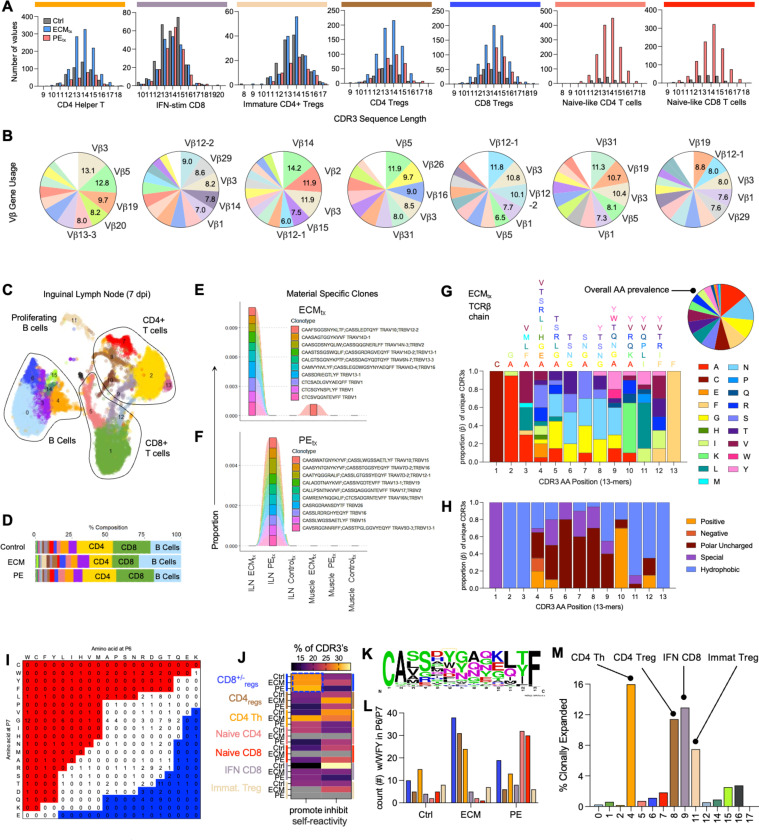
CD8^+/−^Ly49^+^ Killer regulatory cells have unique CDR3 repertoire and are not clonally expanded in response to traumatic muscle injury. (**A**) CDR3 length in main T cell clusters. (**B**) Vβ gene usage in main T cell clusters. (**C**) scRNAseq of ILN 7 dpi. (**D**) Prevalence of clusters across treatment groups in ILN. (**E - F**) Presence of material-specific T cell clones in response to (**E**) ECM and (**F**) PE treated mice. (**G**) Amino acid prevalence in CDR3s of ECM treated mice (13-mers). (**H**) Amino acid (AA) classification in different CDR3 AA positions of ECM treated mice. (**I**) Self-reactivity index (SRI) calculation example, calculation derived from PMID 27348411. (**J**) SRI of T cell clusters across treatment groups. (K) CDR3 enrichment of predicted self-reactive CD8^+/−^Ly49^+^ Tregs. (**L**) Proportion of aromatic residues in P6/P7 of 13-mer CDR3s (TCRβ). (**M**) Clonal expansion of CDR3s in different T cell clusters. Data in A&B are mean ± SEM in n=5 mice. Data in C&D are pooled n=6 mice.

Previous reports of muscle injury using freeze-based injury approaches have reported a clonal, antigen-specific canonical Treg (CD4^+^FoxP3^+^) population that is important in skeletal muscle regeneration^25^. As such, we evaluated the T cell receptor repertoire in traumatic skeletal muscle tissue damage and biomaterial implantation via CDR3 sequencing in scRNAseq data sets of the muscle tissue and draining (inguinal) lymph node (Figure 4). Overall distribution of TCR beta chain CDR3 sequences in the local muscle tissue showed similar medians across treatment groups and cell types with the majority of CDR3 sequences detected being between 13 – 15 amino acids (Figure 4A). Variable beta (Vβ) gene usage was different in different clusters with certain gene segments (ex. Vβ3) being conserved and appearing in top 5 gene segment usage across all cell types tested (Figure 4B). In addition to the local tissue, we sequenced lymphocytes from ILN and identified many of the same cell types that were present in the local tissue (Figure 4C-D). Interestingly, we identified material-specific clones in both the local muscle tissue and the ILN suggesting antigen specificity of responses both to self-antigen as well as exogenous protein derived from proteinaceous biomaterial scaffolds (Figure 4E-F). In reference to the latter case, we were able to identify clones in ECM_tx_ mice that were located both in the muscle tissue and draining lymph nodes (Figure 4E) not present in other treatment groups suggesting their response to a protein derived from the ECM material itself. Locally in the muscle there were T cell CDR3 clones unique to PE_tx_ which may represent a pathologic shift in autoreactive cells as PE does not include any exogenous protein introduction but may alter the recognition of self-protein given its hydrophobic nature (Figure 4F).

A more detailed evaluation of CDR3 sequences showed differences in amino acid enrichment at different positions within the CDR3. When evaluating 13-mer CDR3s (TCRβ chain) we found an overall CA(AA_3–12_)F pattern in the majority of CDR3s evaluated (Figure 4G) with asparagine, glycine, and serine being enriched within the central amino acids. While amino acids on the periphery were more likely to be hydrophobic (positions 2, 3, 13) central amino acids tended towards polar uncharged classifications (Figure 3H). Given these variations, we evaluated potential patterns of amino acids that were previously associated with promoting and inhibiting self-reactivity^26^. We generated self-reactivity indices for all clusters based on their amino acids at positions 6 and 7 of the CDR3 as reported by Stadinski et al (Figure 4I). We found that over 25% of clones detected within the CD8^+/−^Ly49^+^ Treg cluster were identified as “self-reactive” as opposed to other groups with lower indices (Figure 4J). Overall, these predicted self-reactive clones showed an enrichment of aromatic amino acids like tyrosine and tryptophan in positions 6 and 7 (Figure 4K), which was the highest in ECM_tx_ in comparison to control and PE_tx_ injuries (Figure 4L). As there was an enrichment of predicted self-reactive clones in the CD8reg cluster, we then explored clonal expansion among different cell populations (Figure 4M). In response to traumatic physical tissue injury there was clonal expansion of CD4 Treg (brown, cluster 8) and immature CD4 Treg (beige, cluster 11) T cells as was reported in cryoinjury. We also observed clonal expansion of helper CD4+ T cells (orange, cluster 4) and canonical interferon-stimulated CD8+ cytotoxic T cells (purple, cluster 9). In contrast, the CD8^+/−^Ly49^+^ Treg cluster was not clonally expanded representing a non-antigen-specific response.

### Preferential recruitment of B2 B cells to PE treated injuries causes expansion of material-specific potentially autoreactive BCR clones

**Figure 5 |:**
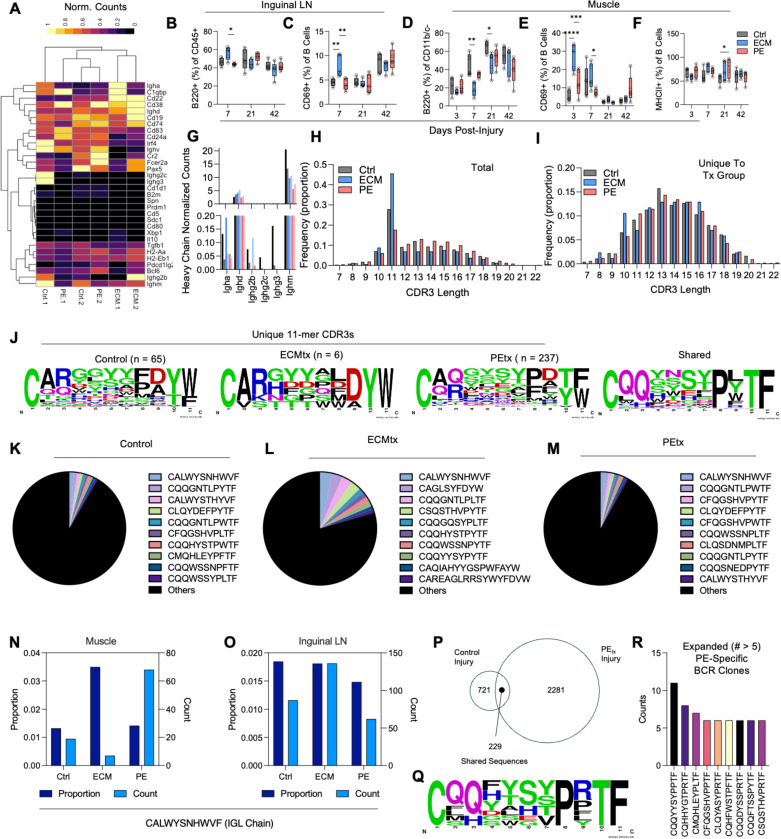
Preferential recruitment of B2 B cells to the local tissue of PE treated injuries causes expansion of material-specific potentially autoreactive clones. (**A**) Normalized gene expression within B cell scRNAseq clusters identifying B1-like (cluster 2) and B2-like (cluster 1) B cells. (**B – F**) Flow cytometric analysis of B cells in (**B - C**) inguinal lymph node (ILN) and (**D – F**) muscle tissue at 7 – 21 dpi. (**B**) B cell prevalence in ILN (**C**) B cell activation in ILN (**D**) B cell prevalence in muscle (**E**) B cell activation in muscle (**F**) MHCII+ B cells in muscle. (**G**) Normalized gene counts of immunoglobulin heavy chain genes in muscle via scRNAseq. (**H - I**) B cell receptor (BCR) CDR3 length for (**H**) common and (**I**) treatment-unique BCR clones across treatment groups. (**J**) Logo plots of amino acid enrichment in treatment-unique and shared BCR clones. (**K - M**) Prevalence of top BCR clones in (**K**) Control (**L**) ECM_tx_, and (**M**) PE_tx_ injuries. (**N - O**) Proportion and count of B cells that are top detected clone CALWYSNHWVF across treatment groups in (**N**) muscle and (**O**) ILN. (**P**) Shared and unique clones in control and PE_tx_ injuries. (**Q**) Logo plot of PE_tx_-unique BCR clones. (**R**) Expanded PE_tx_-specific BCR clones. Data in B-I are mean ± SEM in n=5 mice.

With the local tissue expansion of B cells seen in the PE_tx_ muscle (Figure 3A-B) and regional expansion in the draining lymph node of ECM_tx_ mice (Figure 4C-D) in scRNAseq we wanted to evaluate the potential pathogenic nature of local B cell populations in PE_tx_ mice. As such, we evaluated the phenotype of and clonality (CDR3 sequences) of B cells within the local tissue and ILN of injured mice (Figure 5). Within the muscle two populations of B cells were detected correlating with B1 (Figure 3A, Cluster 2, Yellow) and B2 B cells (Cluster 1, Green) (Figure 5A). Via flow cytometry we confirmed the enrichment of B cells in the inguinal lymph nodes of ECM_tx_ mice at 7dpi (Figure 5B, blue bars). These B cells had significantly higher levels of the early activation marker CD69 suggesting that not only were they present but more activated than those in control and PE_tx_ injury (Figure 5C). scRNAseq of the inguinal lymph nodes also showed a proliferating subset of B cells expressing *Mki67* in addition to *Il21, Cd40lg* and *Icos* expressing T follicular helper cells (Tfh) (Figure S5). Within the muscle, the inverse was true with ECM having the lowest prevalence of B cells in agreement with scRNAseq data (Figure 5D). Though lower in prevalence those that were present in ECM_tx_ wounds did display higher activation especially earlier in response to injury (Figure 5E). Roughly 50% of B cells were MHCII+ in all treatment groups present, this peaked between 7 to 21 dpi (Figure 5F).

Transcriptionally, these B cells in muscle expressed transcripts encoding more immature IgM and IgD heavy chains with ECM_tx_ expressing some transcripts for IgA correlating with type-2 immune responses in these mice (Figure 5G). Among total CDR3 sequences detected for B cell receptors, the peak frequency was detected in a 11 amino acid CDR3 sequence length (Figure 5H); however, for unique CDR3s to each treatment group, these had a broader distribution largely ranging between 11 and 17 amino acids long (Figure 5I). When comparing amino acid prevalence in shared and unique CDR3s (11-mers), we found that unique CDR3s tended towards a CA(AA_3_9)YW pattern whereas shared CDR3s fell into a CQQ(AA_4–7_)PXTF sequence pattern (Figure 5J). Interestingly, when evaluating the top sequences present in overall proportion of B Cell CDR3s we found the highest prevalent clone (CALWYSNHWVF) was conserved across all 3 treatment groups likely correlating with a self-reactive but non-pathogenic clone (Figure 5K-M). This clone represented the largest fraction of B cell sequences locally in the ECM_tx_ muscle tissue (Figure 5N, dark blue) but the PE_tx_ mice had the largest count of these B Cells (Figure 5N, light blue). In the inguinal lymph node, the fraction of B cells that were this clone were highest in Control and ECM_tx_, with the count highest in ECM_tx_ correlating with peripheral expansion of these cells in response to ECM (Figure 5O). When evaluating the sequence differences between control and PE_tx_ injury, we found shared and expanded sequences induced by the non-proteinaceous hydrophobic plastic (Figure 5P-R). These shared sequences followed the same CQQ(AA_4_7)PXTF previously mentioned (Figure 5P-Q). When evaluating the unique and expanded clones present in PE_tx_ muscle, we found a number that could represent pathogenic auto-reactive clones (Figure 5R).

### CD8^+/−^Ly49+ killer regulatory T cells co-localize with cDC1s in mice and selectively proliferate in vitro with damage associated molecular patterns

**Figure 6 |:**
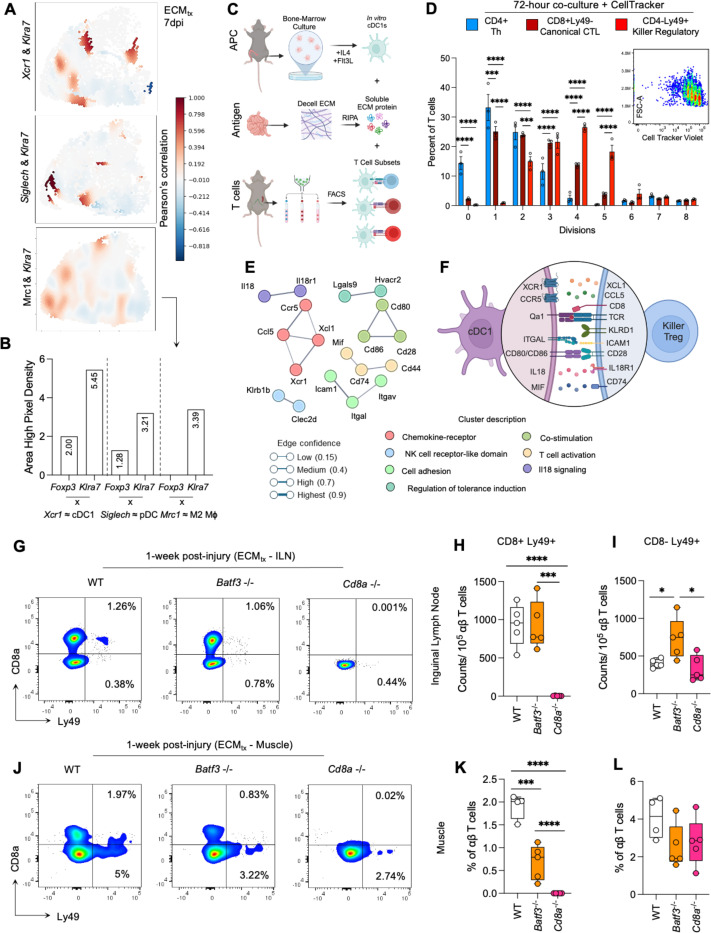
CD8^+/−^Ly49^+^ Killer regulatory T cells co-localize with cDC1s in mice and selectively proliferate in vitro with DAMP stimulation. (**A**) Spatial coincidence (Pearson’s Correlation) of indicated gene pairs within muscle tissue of ECM_tx_ mice at 7dpi. (**B**) Quantification of area of positive (>0.2) correlated gene coincidence of gene pairs representing cDC1s, pDCs, CD4 canonical Tregs, CD8^+/−^Ly49^+^ Tregs, and M2-like macrophages. (**C**) Workflow for cDC1-Killer T reg co-culture for proliferation analysis, Created in BioRender. (**D**) Quantification of cell proliferation by CellTracker after 72 hours of co-culture with different cDC1-lymphocyte pairs. (**E**) Protein-protein interactions and gene ontology of enriched cDC1 and Ly49+ Treg markers via StringDB^35^.(**F**) Schematic depicting putative pathways of communication between cDC1s and CD8^+/−^Ly49^+^ Tregs.(**G-H**) Flow cytometry analysis and quantitation of CD8+ Ly49+ T cells and CD8- Ly49+ T cells within inguinal lymph nodes (**G-I**) and muscle tissue (**J-L**) of cDC1-deficient (*Batf3*^−/−^) and CD8-deficient (*Cd8a*^−/−^) mice at 7 dpi with ECM_tx_, Data are mean ± IQR n=5. Data in B are mean ± SEM in n=3 mice. Data in G-L are mean ± SEM in n=5 mice.

As antigen presentation and recognition are cell-cell contact mediated, these cells must be significantly co-localized in the tissue in comparison to other cell types. Indeed, when we looked at spatial expression of *Xcr1* and *Klra7* which are highly expressed in cDC1s and CD8 Tregs, respectively, we observed spatial co-incidence hot spots in muscle tissue at 7dpi (Figure 6A-B). Unsurprisingly, spatial co-localization was also apparent with markers expressed within the same cell such as *Ikzf2* and *Klra7*. Critically, the co-localization of *Xcr1* and *Klra7* was not due to abundance alone, since co-localization was not evident with either *Mrc1* or *Siglech* expression which are characteristic markers of abundant subsets of macrophages (M2 macrophages) and DCs (plasmacytoid DCs) in the muscle (Figure 6B). Additionally, spatial expression of characteristic markers of CD8^+/−^Ly49^+^ Tregs such as *Xcl1*, *Ccl5*, *Gramd3*, *Il2rb* and *Klrb1c* was greater at the injury and implantation site (Figure S6A).

Having observed co-localization, we next asked if the interaction between cDC1s and CD8^+/−^Ly49^+^ Tregs gave rise to CD8^+/−^Ly49^+^ Treg expansion which is a characteristic response to antigen presentation events *in vitro*. To test this, we co-cultured bone marrow derived, Flt3-L induced cDC1s pulsed with ECM proteins and CD8^+/−^Ly49^+^ Tregssorted from inguinal lymph nodes and spleens of mice with ECM_tx_ injuries (Figure 6C). Cell trace violet dilutions of CD4+Ly49- T cells, CD8+Ly49- T cells and CD8^+/−^Ly49^+^ T cells showed selective proliferation of CD8^+/−^Ly49^+^ T cells 72 hours after co-culture (median = 2 divisions vs 4 divisions, p<0.0001; Figure 6D). Further, in *Rag1*^−/−^ mice cDC1 recruitment to the muscle was significantly diminished suggesting interdependence of T cells and cDC1s for proliferation and chemotaxis (Figure S6B). Next, to identify potential pathways of cDC1- CD8^+/−^Ly49^+^ Treg crosstalk, we extracted highly expressed markers and used those and inputs for molecular pathway analysis using the STRING database (Figure 6E). We found enrichment of IL-18 signaling, T cell activation, and chemokine-receptor interactions (Figure S6C). Overall, through these databases and CellChat analyses (Figure S7A-B), we identified 9 distinct communication networks between cDC1s and CD8 Tregs which could result in priming and downstream effector functions (Figure 6F). Lastly, to test the effect of cDC1 depletion on killer Tregs, and to investigate downstream events in mice lacking killer Tregs, we performed VML and implanted ECM in *Batf3*^−/−^ and *Cd8a*^−/−^ mice and evaluated the prevalence of CD8+ and CD8+/−Ly49+ Tregs in the draining lymph node and muscle of ECM_tx_ muscle injuries at 7 dpi (Figure 6G-L). There was no significant difference in CD8+/−Ly49+ Tregs in *Batf3*^−/−^ mice in the ILN with a small but significant increase of CD8^+/−^Ly49^+^ Tregs (p=0.039 for WT vs *Batf3*^−/−^ and p=0.014 for *Batf3*^−/−^ vs *Cd8a*^−/−^ Figure 6H-I). As expected CD8^+/−^Ly49^+^ Tregs were absent in *Cd8a*^−/−^ mice, with CD8^+/−^Ly49^+^ being unchanged. Interestingly, in the muscle there was a significant decrease in CD8+ killer Tregs in *Batf3*^−/−^ mice (p<0.001 vs WT) (Figure 6J-K) with a small but insignificant decrease in CD8^+/−^Ly49^+^ Tregs (Figure 6L). As with the ILN, CD8^+/−^Ly49^+^ Tregs were completely absent in *Cd8a*^−/−^ mice (Figure 6K), and a small but insignificant decrease in CD8^+/−^Ly49^+^Tregs was detected (Figure 6L). In agreement with previous reports of compensating regulatory mechanisms, CD4+FoxP3+ Tregs were significantly elevated in *Batf3*^−/−^ and *Cd8a*^−/−^ mice which received ECM_tx_ at 7dpi (p=0.001 for WT vs *Batf3*^−/−^ and p=0.024 for WT vs *Cd8a*^−/−^; Figure S6D). Lastly, although not statistically significant, HELIOS expression was elevated in *Batf3*^−/−^ mice as compared to *Cd8a*^−/−^ mice and WT controls (Figure S6E). These data suggest that cDC1s prime CD8^+/−^Ly49^+^ Tregs in the muscle which results in expansion of CD8^+/^Ly49^+^ Tregs.

### Accumulation of naïve T cells in the muscle is mitigated due to selective pruning by CD8+ Tregs

**Figure 7 |:**
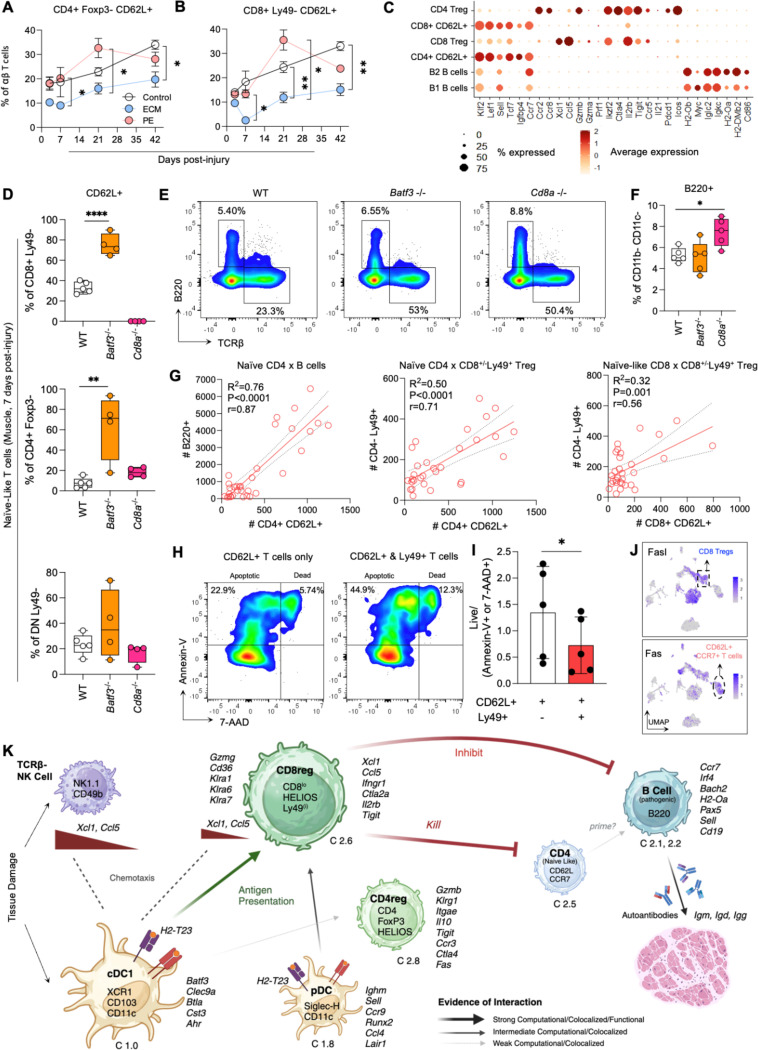
Accumulation of autoreactive-like B2 B Cells is mitigated by selective pruning of naïve helper T cells in the muscle by CD8^+/−^Ly49^+^ Killer Tregs. (**A** and **B**) Accumulation of naïve CD62L^+^FoxP3^-^HELIOS^-^ CD4^+^ (**A**) and CD8^+^ T cells (**B**) in muscle tissue over time via flow cytometry (**C**) Differentially expressed genes in select T and B cell clusters in the muscle at 7 dpi.(**D - F**) Flow cytometric quantitation of (**D, E**) naïve T cell subsets and (**E, F**) B cells in WT, *Batf3*^−/−^ and *Aire*^−/−^ mice. (**G**) Correlated cell abundance between indicated subsets, trendline = simple linear regression ± 95% CI. (**H**) Representative flow cytometry plots and (**I**) quantification (ratio of live to apoptotic/dead) of apoptosis (Annexin V) and cell death (7-AAD) with *in vitro* co cultures of CD8^+/−^Ly49^+^ Treg and CD62L+CD4+ naïve helper T cells and monocultures of CD62L+ T cells. (**J**) *Fasl* and *Fas* expression in lymphocyte cell clusters from scRNAseq, UMAP with overlaid normalized gene expression (**K**) Schematic depicting overall mechanism of cDC1 stimulation of CD8^+/−^Ly49+ Tregs, cells labeled as C#A.B with A = dendritic cell dataset (1) or lymphocyte data set (2) and B = cluster number within the dataset, Created in BioRender. Data in A-I are mean ± SEM, n≥3 mice. Data in J are a pooled n=10 mice.

Extralymphoid tissues do not experience naïve T cell accumulation under homeostasis^8^. Cumulative recruitment of naïve T cells to non-lymphoid organs is associated with autoimmunity. Specifically, CCR7 and CD62L expressing naïve T cells are known to aggregate within non-lymphoid tissues and form tertiary lymphoid structures. Furthermore, previous reports show that B cells within these structures behave similarly to B cells within germinal centers and potentiate autoimmunity. In mice which received controls and PE_tx_, but not ECM_tx_, we observed that CD62L+ T cells of both CD4 (p=0.02 ECM_tx_ vs PE_tx_ at day 21 post-injury and p=0.025 for control vs ECM_tx_ at day 42 post-injury) and CD8 (p=0.01 ECM_tx_ vs PE_tx_ at day 21 post-injury) lineage accumulate over time (Figure 7A-B). These findings were corroborated by scRNAseq which showed low expression of *Cd44* coupled with high expression of *Ccr7*, *Sell, Klf2, Tcf7* and *Fas* (Figure 7C).

In mice lacking cross-presentation and cDC1s (*Batf3*^−/−^), both CD4+CD62L+ (p=0.0019 vs WT) as well as CD8+CD62L+ (p<0.0001 vs WT) T cells were elevated at 7dpi (Figure 7D). In addition to changes in naïve T cells in the absence of cDC1s, B cell abundance in the muscle was significantly elevated within *Cd8a*^−/−^ mice which received ECM_tx_ (p=0.039 WT vs *Cd8a*^−/−^ Figure 7F). While the *Batf3*^−/−^ have elevated naïve T cells (but not significant in *Cd8a*^−/−^) and *Cd8a*^−/−^ having elevated B cells (but not significant in *Batf3*^−/−^) this suggests that both cell types are needed for this process and like many immune cascades there are redundancies built into the system. In WT mice, naïve CD4+ T cell abundance strongly correlated with B cell abundance (Pearson r=0.87) within the muscle (Figure 7G). Furthermore, both CD4+ (r=0.71) and CD8+ (r=0.56) naïve T cell abundance correlated with CD8^+/−^Ly49^+^ T cell abundance but not as strongly as the correlation with B cells.

To test whether CD8^+/−^Ly49^+^ Tregs can target naïve T cells, we isolated CD62L+CCR7+ T cells from the muscle of mice which received PE_tx_ at 7dpi. Concurrently, we isolated CD8^+/−^Ly49^+^ T cells from the muscle of mice which received ECM_tx_ at 7dpi. We expanded these cells *in vitro* for 7 days and then co-cultured for 16 hours. We stained all cells with Annexin-V and 7-AAD to detect apoptotic and dead cells (Figure 7H). Ly49+ T cells selectively killed T cells that regained CD62L expression suggesting a capacity for targeted elimination (p=0.013; Figure 7I). Given the lack of activation molecules on these cells, targeting these cells would require complimentary binding mechanisms such as the Fas-FasL pathway. Interestingly, *Fasl* expression was highest on CD8^+/^Ly49^+^Tregs and not CD4+ T regs and *Fas* was strongly abundant on naïve T cells (Figure 7J).

Our data suggest a rapid tolerance mechanism based on cDC1-mediated activation of killer CD8^+/^Ly49^+^ Tregs to prune naïve T cells and prevent B cell accumulation in the injury site. This mechanism is subverted by particles of the hydrophobic plastic PE, which leads to naïve T cell accumulation in the muscle and B cell abundance. By enriching cDC1s and CD8^+/−^Ly49^+^ Tregs, pro-healing ECM biomaterials mitigate naïve T cell retention in tissue and prevents B cell driven autoimmunity (Figure 7K).

## DISCUSSION

Here, we described a regulatory pathway in response to traumatic injury that forms the basis of a quick-acting innate-like response to prevent autoimmunity in times of instantaneous, significant inflammation and tissue damage. Traumatic injury causes a rapid release of self-antigen into a strongly inflammatory environment that is susceptible to infection and foreign body presence from environmental debris.

We found that after recruitment of cross-presentation capable cDC1’s to the injury microenvironment by NK cells, there is a damage-associated proliferation of CD3^+^TCRβ^+^HELIOS^+^Ly49(in)^+^CD8^+/−^ killer-like regulatory T cells (CD8^+/−^Ly49^+^ Tregs). These killer-like regulatory cells are preferentially primed by cDC1s in the presence of damage associated molecular patterns (DAMPs) and kill naïve CD4 and CD8+ T cells that accumulate in pathogenic implants. Absence of cDC1s and CD8+ T cells through genetic knockdown correlates with increased naïve T cell and B cell presence, respectively. B cell receptor repertoire identifies potential pathogenic clones that accumulate in PE_tx_ injuries that may be associated with production of autoantibodies. Indeed, cDC1-deficient mice have a transcriptional phenotype that mirrors autoimmune mice that lack the transcription factor Aire, needed for central self-tolerance establishment. Given the highly variable T cell receptor repertoire detected in the killer-like regulatory T cell population in comparison to conventional CD4+ Tregs, this might represent a rapid immunologic self-tolerance pathway evolved to mediate these instances of extreme tissue damage.

Conventional Tregs (CD4+FoxP3+) are potent suppressors of antigen stimulated T cell proliferation. However, in our dataset, we find that T cells which express *Ccr7*, and *Sell* while also lacking expression of *Cd44, Itgae*, granzymes or perforin accumulate within the muscle, a non-lymphoid organ, after tissue damage. Given this lack of antigen exposure and activation, these cells could be evasive to CD4+FoxP3+ Treg driven elimination. Furthermore, accumulation of naïve T cells in non-lymphoid organs is known to be a hallmark of tertiary lymphoid structure formation^8^. We posit that naïve T cell targeting could be a potent mechanism of preventing tertiary lymphoid structure formation in non-lymphoid tissue and CD8^+/−^Ly49^+^ Tregs execute this function upon tissue damage. Additionally, the CD8^+/−^Ly49^+^ T cell population in our animal model, includes a sub-population of NKT-like cells (in addition to the separate NKT cluster) as defined by concomitant expression of *Ncr1* and *Cd3e,* a recently described population of T cell expressing *Cd8a, Ikzf2, and Klra4/6/7,* and *Klrb1c* expressing MAIT cells. These populations of T cells appear to be transcriptionally similar, as reported with MAIT and NKT cells but not CD8+ Tregs^27,28^, beyond identity defining markers raising the possibility that these cells are functionally similar. As such it is possible that CD8^+^HELIOS^+^Ly49^+^ Tregs are invariant like NKT cells and MAIT cells. Investigating the antigen specificity, ontogeny and convergence of the role of CD8^+^HELIOS^+^Ly49^+^ T cells with other Ly49^+^ subsets remains to be understood.

Indeed, other studies have found these regulatory CD8 T cells in mice and humans wherein tissue damage occurs such as lung damage severe COVID19 infections^24^, organ transplant^16,18^, and multiple sclerosis^17^. Other regulatory cell populations, such as canonical CD4+ Tregs have been reported to be critical in repair and regeneration of skeletal muscle^11,12,29^. We propose here that this early self-tolerance pathway is conserved across all situations of tissue damage, working in concert with antigen specific regulatory pathways to prevent pathology and help rebuild damaged tissues. Such pathways could be leveraged not only for therapeutics in regenerative medicine but development of novel medical device coatings and treatments of autoimmune diseases.

### Limitations of the study

Due to the relatively novel nature of this cell population (regulatory CD8^+/−^Ly49^+^ T cells) and limited research conducted to date relative to more common cell types (ex. CD4+ Tregs) technical reagent limitations did not allow for some analyses that would prove useful for confirming some findings including protein-level analyses on spatial distribution of cells within mouse and human histologic specimens. Clinical and ethical limitations in accessing skeletal muscle tissue from human patients in comparison to specimens such as tumors that are readily resected also leave a void in meta-analyses on data that are difficult to obtain.

## MATERIALS AND METHODS

### Preparation of biomaterials

Biomaterials used as implants in mice were prepared as reported previously^13,30,31^. Small intestines obtained from 5 to 6-month-old American Yorkshire pigs were washed with water to remove feces and mechanically scraped to remove cell and mucus layers. The sub-mucosa (SIS) was then cut into 1 to 2-inch segments, washed in sterile deionized water and incubated in 10% Antibiotic-Antimycotic solution overnight at room temperature while stirring to remove any remaining fecal matter and mucosal tissue. Following incubation, SIS was washed with sterile deionized water and incubated at room temperature for 30 minutes in a solution containing 0.1% peracetic acid and 4% ethanol in sterile deionized water. Following this, SIS was subjected to alternating washes in filter sterilized tap water and 1x PBS until pH neutralization. Lastly, SIS was lyophilized for 48 hours and cryogenically milled to obtain a fine powder which was rehydrated to 200 mg/mL with a paste-like consistency before injecting at the site of implantation through a sterile syringe. Polyethylene (PE) particles of 150 μm diameter were subjected to shortwave UV-irradiation for 40 minutes, resuspended in 70% ethanol overnight, transferred to Eppendorf tubes and air dried in a sterile biosafety cabinet.

### Volumetric muscle loss surgery

All mice received bilateral volumetric muscle loss procedures unless noted otherwise. One to three days prior to surgery 6–8 week old C57BL/6 WT (Jackson Laboratory stock # 000664), *Cd8a*^−/−^ (Stock # 002665^32^), *Batf3*^−/−^ (Stock # 013755^33^) and *Aire*^−/−^ (Stock # 004743^34^) mice were cleared of hind limb and abdominal fur by shaving with electric razor followed by depilatory cream application. Anesthesia was first induced in a chamber supplied with 4% isoflurane and maintained with a nosecone supplied with 2% isoflurane supplemented with oxygen at a flow rate of 200 mL/min. Skin overlying the quadriceps was sterilized three times with betadine and isopropanol alternatingly in a circular pattern working from the incision site outward. Following this, a 1cm surgical incision into the skin and fascia was made to isolate the quadriceps muscle and a 3mm defect was created centrally using sterile surgical scissors. The wound was then backfilled with either ECM, PE or no biomaterial as a control. All mice were monitored until reported endpoints. The protocol (NIH Clinical Center ACUC Protocol NIBIB23–01) was reviewed approved by the National Institutes of Health Scientific Review and Animal Care and Use Committees.

### Isolating dendritic cells and lymphocytes

C57BL/6 WT mice which received biomaterials and control injuries were euthanized at 7 days post-injury via carbon dioxide asphyxiation followed by cervical dislocation. Quadriceps along with biomaterials were dissected, minced, digested in a buffer comprising 0.25 mg/mL Liberase TM and 0.2 mg/mL DNAse I in serum-free Dulbecco’s Modified Eagle Medium (DMEM) for 1 hour, and passed through a 70 μm cell strainer. Inguinal lymph nodes were mashed and passed through a 70 μm cell strainer and washed with 1x PBS. The resulting single cell suspensions were stained with 7-AAD for 10 minutes and a surface marker cocktail to isolate dendritic cells from muscle tissue and lymphocytes from muscle tissue and inguinal lymph nodes respectively. Antibodies identifying surface markers for DC, B and T cells isolation are in Supplemental Table S2. Cells were sorted using a BD FACSMelody sorter into a buffer comprising 50% FBS and 25mM HEPES in 1x PBS. For single cell RNA sequencing analysis, cells were centrifuged at 400 xg at 4 C for 5 minutes, resuspended in Recovery^™^ Cell Culture Freezing Medium (ThermoFisher Scientific), frozen overnight at –80°C in a slow cooling apparatus and transferred to a liquid nitrogen tank until further analysis. Cells were shipped on dry ice to Azenta Life Sciences for single cell RNAseq.

### Flow cytometry preparation and data analysis

Whole blood leukocytes, quadriceps muscle, and inguinal lymph nodes (ILN) were obtained from mice at predetermined endpoints using standard dissection and isolation procedures. Muscle tissue was minced and either digested in 0.05% Collagenase IV and 0.2 mg/mL DNAse I to stain for dendritic cell markers or directly mechanically dissociated on a 70 μm cell strainer and washed with a buffer comprising 1% BSA and 2 mM EDTA to stain for enzyme sensitive lymphocyte markers. Peripheral blood was collected through submandibular bleeding in 5mM EDTA in 1x PBS and red blood cells (RBC) were lysed using eBioscience 1x RBC Lysis buffer (Multi-species) before analysis. Lymph nodes were mechanically dissociated through a 70 μm cell strainer and washed with a buffer comprising 1% BSA and 2 mM EDTA (wash buffer). Resulting single cell suspensions were washed in 1x PBS and resuspended in 200 μL of Live/Dead Blue (1:1000 dilution) for 20 minutes at 4°C. Cells were then washed, resuspended in 50 μL of solution containing TruStain Monocyte block (1:10 dilution) and TruStain Fc block (1:100 dilution). Separate panels for dendritic cell markers and lymphocyte markers were used to analyze samples (Supplemental Table S2). Using these panels, cells were stained for 1 hour at 4°C, washed in wash buffer at 400 xg for 5 minutes at 4°C and fixed using True-Nuclear Transcription Factor kit (Biolegend). Lymphocytes were washed in a permeabilization buffer (True-Nuclear Transcription Factor kit (Biolegend)) at 400 xg for 5 minutes at room temperature and stained overnight for intracellular markers at 4°C. Cells were washed at 400 xg for 5 minutes at 4°C and resuspended in wash buffer prior to analysis on a 5 laser Cytek Aurora spectral flow cytometer. Data were unmixed via single-spectra controls using SpectroFlo (v3.2.1) then unmixed data were analyzed via FlowJo (v10.10.0). Resulting data were analyzed on GraphPad Prism v10.3.1.

### Single cell RNA sequencing raw data processing, analysis and visualization

Sequenced reads were aligned, and feature-barcode matrices were generated using Cell Ranger. Matrices were read into R and the DropletsUtils package was used to filter out empty droplets and generate final filtered counts for analysis. The Seurat package (v4.3.0) was used to filter out cells containing > 20% mitochondrial or ribosomal counts for each treatment group. Finally, samples from all treatment groups were merged to create a unified Seurat object. Following the standard Seurat pipeline, Uniform Manifold Approximation and Projection (UMAP) clusters were generated after logarithmic normalization (LogCP10K), data scaling and top 2000 features selected using variance stabilizing transform method for principal component analysis (PCA) retaining the top 50 principal components for computing nearest neighbors. Cluster identity was assigned using either canonical or published literature. Latent time analysis was run in Python v3.8.18 using the “SpatialCorr” and “Scanpy” libraries.

For mined mouse data, GSE200289^22^ and GSE142471^21^ were retrieved from GEO and subclustered prior to display of CD8reg gene signature on T cell sub-cluster. For mined human scRNAseq data, GSE143704^23^ of human skeletal muscle tissue was subset to evaluate CD3+ T cells. Within this subset, cells expressing genes encoding KIRs and IKZF2 were displayed using GraphPad Prism v10.3.1.

### T and B cell receptor sequencing data analysis and visualization

T and B cell receptor data was analyzed using the “cellranger vdj” pipeline. FASTQ files were input via command line to run “cellranger vdj” and filtered and annotated outputs were used for downstream analysis. For visualizing gene usage, the Vβ segments were filtered and displayed using a pie chart. Clonotypes generated using cellranger were analyzed using the “immunarch” package in R (4.2.1). Clonotypes were tracked between muscle and lymph node datasets using the “trackClonotypes” function based on amino acid sequence matching. CDR3 length and hydrophobicity scoring were analyzed using Excel with relevant CDR3 sequence lengths as stated in the text, then data were displayed using GraphPad Prism v10.3.1. Logo plots were generating using WebLogo (UC Berkeley).

### Spatial transcriptomics data analysis and visualization

5 μm cross sections of quadriceps were taken from mice which received VML and biomaterial implants at 1 week post injury. Sections were H&E stained and imaged using a Leica brightfield microscope for mapping transcripts to tissue location. Spatial transcriptomic sequencing was carried out using a 10X Visium instrument at the NICHD molecular genomics core facility. The standard Space Ranger workflow was used to generate gene expression matrices using the mm10 reference transcriptome. Outputs were subsequently imported into an R Studio workspace to run the Seurat pipeline for analyzing spatial datasets. Counts were normalized using the function “SCTransform” and gene expression was visualized using the “SpatialFeaturePlot” function. To plot signatures from single cell RNA sequencing datasets, references were normalized using the “SCTransform” function and an anchor-based integration method was used to generate prediction scores for each spatial spot. Additionally, we also generated module scores for gene sets containing enriched genes in specific clusters and visualized the score using “SpatialFeaturePlot”.

### Bulk RNA sequencing data analysis and visualization

Bulk RNA sequencing data analysis was performed as previously reported^31^. Briefly, whole quadriceps muscles were flash-frozen on dry ice for bulk RNA isolation and sequencing to Azenta GeneWiz. Data analysis was performed on the paired end reads from 12 independent samples. Pseudoaligment was performed using Kallisto to map reads to a publicly available reference transcriptome (Ensemble GRCm39). The Ensemble Mus musculus v79 annotation database mapped transcripts to gene IDs. EdgeR package was used to determine counts per million and subsequently filtered by counts per million > 1 for at least 3 samples and log2 transformed. Data was normalized using the Trimmed Mean of M-values method. Differential expression was visualized using standard functions to generate fold change plots for each pairwise comparison. Gene set enrichment analysis (GSEA) was carried out in R using the “GSEABase” package and molecular signatures gene set collection.

### Protein isolation and bone marrow-derived cDC1 generation

Decellularized ECM and muscle tissue were homogenized at 5000 rpm for 30 seconds using a handheld homogenizer (Fisherbrand homogenizer 850) in RIPA buffer (ThermoFisher) containing protease inhibitors (Pierce, ThermoFisher). Isolates were diluted, resuspended in sterile deionized water and quantified using Pierce BCA assay following manufacturer’s instructions. Protein solutions were stored at –20 C until further use. Bone marrow cells were isolated from the femur bones of healthy 8-week-old C57BL/6 mice. Cells were cultured for 9 days in media containing GM-CSF (5 ng/mL), FLT3L (150 ng/mL) in complete RPMI 1640 medium (10% FBS, 1% Penicillin/Streptomycin) with glutamine. Weakly adhered and floating cells were collected, and media was replenished on days 3 and 6 of culture. Resulting cells were matured overnight by lipopolysaccharide (LPS) stimulation (50 ng/mL) and loaded with protein isolated from ECM and muscle tissue (1 μg/mL).

### In vitro T cell stimulation and proliferation

Ly49+ CD8+ T cells were isolated flow cytometrically as described above. T cells were washed at 400 xg for 5 minutes at 4 C and resuspended in 1x PBS containing cell trace violet stain (1:1000 dilution) and incubated in a water bath at 37 C for 20 minutes. T cells were washed in complete media and resuspended in complete RPMI 1640 medium (10% FBS, 1% Penicillin/Streptomycin) supplemented with IL-2 (1ng/mL), GM-CSF (5 ng/mL), FLT3L (100 ng/mL) and 50 μM β-mercaptoethanol. T cells were cultured with induced cDC1s loaded with either protein isolated from ECM. A 1:2 ratio of APCs:T cells was used and cell culture was carried out for 3 days prior to analysis on Cytek Aurora flow cytometer.

### In vitro apoptosis and cell death detection

CD4-Ly49+ T cells and CCR7+CD62L+ T cells were isolated at 7 days post-injury from muscle tissue of mice which received ECM_tx_ and PE_tx_ respectively. Cells were stimulated with soluble CD3ε antigen (1 μg/mL) for 24 hours in the presence of recombinant IL-2. Following stimulation, cells were washed with 1x PBS and replated in complete RPMI 1640 media containing recombinant IL-2 (10 ng/mL) for 6 additional days. Media was replenished every 48 hours. CCR7+CD62L+ T cells were then co-cultured with CD4-Ly49+ T cells for 16 hours. Cells were pelleted, washed and stained with conjugated antibodies against Annexin-V (eFluor-450), CD62L(APC-Cy7), CCR7(APC), Ly49(PE) in Annexin-V binding buffer for 30 minutes at room temperature followed by washing and staining with 7-AAD for 30 minutes at room temperature and analyzed on a Cytek aurora flow cytometer.

### Immunohistochemistry and Immunofluorescence

Human and mouse tissue samples taken were fixed in neutral buffered formalin for 48 hours prior to sequential dehydration in 70%, 80%, 95% and 100% ethanol. Samples were then cleared in xylene and paraffin embedded before sectioning at a thickness of 5 μm. Sections were heated to 60 ⁰C overnight, cooled to room temperature, rehydrated, incubated in boiling citrate antigen retrieval buffer for 20 minutes and cooled to room temperature. All sections were blocked using a solution containing 4% normal goat serum, 1% bovine serum albumin and 0.05% Tween-20 in 1x PBS for 1 hour. Following this, samples were incubated with primary antibodies (Rat anti Mouse B220; BioLegend and Rabbit anti Mouse CD3e; Abcam) overnight at 4 ⁰C in a humidity chamber. After three washes in 0.05% Tween-20 in 1x PBS, samples were incubated with secondary antibodies (Donkey anti Rat Alexa Fluor-594; Invitrogen and Goat anti Rabbit Alexa Fluor-488; Invitrogen) for 90 minutes at room temperature. Autofluorescence was quenched in all samples using Vector True View autofluorescence quenching kit (Vector Labs) following manufacturer’s instructions. Finally, samples were counter stained with DAPI, washed in DI water, mounted and imaged using an EVOS M5000 microscope. Images were adjusted for brightness and contrast in FIJI (ImageJ2, Version 2.14.0/1.54f) using primary delete controls to set thresholds.

### Statistical analysis and quantification.

Statistics were computed in R (v4.2.1) and GrpahPad Prism (v10.4.0). Bulk RNA-sequencing data was analyzed using empirical Bayes statistics for differential expression method using the R package “limma”. To compare various lymphocyte subsets using flow cytometry, unpaired T-tests were used to compare knock out mice to WT controls. In the co-culture assay, to compare means of the two indicated populations, a one-tailed paired T-test was used. One-way ANOVA tests with Tukey’s correction for multiple comparisons were used to compare various populations across treatment groups within WT mice and to compare populations across various knock out mice with ECM_tx_. Where indicated, normality testing was performed using the Shapiro-Wilk test. Datasets which failed normality testing were analyzed non-parametrically. Muscle samples with very low T cells yield (less than 500 cells) were omitted from analysis. None of the samples from the lymph nodes were omitted. Transcriptomic signature scores were calculated using the “AddModuleScore” function within Seurat using the genes represented in Table S1. To compare accumulation of naïve T cells in the muscle across treatment groups via flow cytometry, a mixed effects model approach was used. Gene ontology was obtained from the STRING database and gene set enrichment analysis was performed using the “GSEABase” and “msigdbr” packages within R. For correlation testing, simple linear regression analysis was done in GraphPad Prism (v10.4.0). Unless otherwise indicated, ^ns^p>0.05, *p<0.05, **p≤0.01, *** p≤0.001 and **** p≤0.0001. “r” values represent Pearson’s correlation coefficient.

## RESOURCE AVAILABILITY

### Lead contact

Requests and inquiries on reagents and sources will be fulfilled by the lead contact- Dr. Kaitlyn Sadtler (kaitlyn.sadtler@nih.gov).

### Materials availability

The materials, sources and pertinent information required to reproduce data is available within this manuscript.

## Supplementary Material

Supplement 1

## Data Availability

Raw data files and code used to analyze data and generate figure panels will be made publicly available upon acceptance of the manuscript.

## References

[1] Matzinger P. Tolerance, danger, and the extended family. Annual review of immunology 12, 991–1045 (1994). 10.1146/annurev.iy.12.040194.005015 8011301

[2] Medzhitov R. & Janeway C. A. Jr Decoding the patterns of self and nonself by the innate immune system. Science 296, 298–300 (2002). 11951031 10.1126/science.1068883

[3] Franks A. L. & Slansky J. E. Multiple associations between a broad spectrum of autoimmune diseases, chronic inflammatory diseases and cancer. Anticancer research 32, 1119–1136 (2012). 22493341 PMC3349285

[4] Hileman C. O., Malakooti S. K., Patil N., Singer N. G. & McComsey G. A. New-onset autoimmune disease after COVID-19. Front Immunol 15, 1337406 (2024). 10.3389/fimmu.2024.1337406 PMC10883027 38390319

[5] Cohen Tervaert J. W., Colaris M. J. & van der Hulst R. R. Silicone breast implants and autoimmune rheumatic diseases: myth or reality. Curr Opin Rheumatol 29, 348–354 (2017). 10.1097/bor.0000000000000391 28379860

[6] Cohen Tervaert J. W. Autoinflammatory/autoimmunity syndrome induced by adjuvants (Shoenfeld’s syndrome) in patients after a polypropylene mesh implantation. Best Pract Res Clin Rheumatol 32, 511–520 (2018). 10.1016/j.berh.2019.01.003 31174820

[7] Rosenblum M. D., Remedios K. A. & Abbas A. K. Mechanisms of human autoimmunity. The Journal of clinical investigation 125, 2228–2233 (2015). 25893595 10.1172/JCI78088 PMC4518692

[8] Ruddle N. H. High Endothelial Venules and Lymphatic Vessels in Tertiary Lymphoid Organs: Characteristics, Functions, and Regulation. Front Immunol 7, 491 (2016). 10.3389/fimmu.2016.00491 27881983 PMC5101196

[9] Horsley V., Jansen K. M., Mills S. T. & Pavlath G. K. IL-4 acts as a myoblast recruitment factor during mammalian muscle growth. Cell 113, 483–494 (2003). 12757709 10.1016/s0092-8674(03)00319-2

[10] Sadtler K. Developing a pro-regenerative biomaterial scaffold microenvironment requires T helper 2 cells. Science 352, 366–370 (2016). 27081073 10.1126/science.aad9272 PMC4866509

[11] Gama J. F. Role of regulatory T cells in skeletal muscle regeneration: A systematic review. Biomolecules 12, 817 (2022). 35740942 10.3390/biom12060817 PMC9220893

[12] Panduro M., Benoist C. & Mathis D. Treg cells limit IFN-γ production to control macrophage accrual and phenotype during skeletal muscle regeneration. Proceedings of the National Academy of Sciences 115, E2585–E2593 (2018). 10.1073/pnas.1800618115 PMC5856564 29476012

[13] Lokwani R. Pro-regenerative biomaterials recruit immunoregulatory dendritic cells after traumatic injury. Nature Materials (2023). 10.1038/s41563-023-01689-9 PMC13504775 37872423

[14] Sadtler K. Divergent immune responses to synthetic and biological scaffolds. Biomaterials 192, 405–415 (2019). 30500722 10.1016/j.biomaterials.2018.11.002

[15] Jacobs J. J., Hallab N. J., Urban R. M. & Wimmer M. A. Wear particles. JBJS 88, 99–102 (2006). 10.2106/JBJS.F.00102 16595453

[16] Choi J. Y. Regulatory CD8 T cells that recognize Qa-1 expressed by CD4 T-helper cells inhibit rejection of heart allografts. Proc Natl Acad Sci U S A 117, 6042–6046 (2020). 10.1073/pnas.1918950117 32111690 PMC7084119

[17] Saligrama N. Opposing T cell responses in experimental autoimmune encephalomyelitis. Nature 572, 481–487 (2019). 10.1038/s41586-019-1467-x 31391585 PMC7145319

[18] Stocks B. T., Wilson C. S., Marshall A. F., Brewer L. A. & Moore D. J. Host Expression of the CD8 Treg/NK Cell Restriction Element Qa-1 is Dispensable for Transplant Tolerance. Sci Rep 7, 11181 (2017). 10.1038/s41598-017-11780-2 PMC5593978 28894277

[19] Uss E. CD103 is a marker for alloantigen-induced regulatory CD8+ T cells. The Journal of Immunology 177, 2775–2783 (2006). 16920912 10.4049/jimmunol.177.5.2775

[20] Xystrakis E. Identification of a novel natural regulatory CD8 T-cell subset and analysis of its mechanism of regulation. Blood 104, 3294–3301 (2004). 15271801 10.1182/blood-2004-03-1214

[21] Haensel D. Defining Epidermal Basal Cell States during Skin Homeostasis and Wound Healing Using Single-Cell Transcriptomics. Cell Rep 30, 3932–3947.e3936 (2020). 10.1016/j.celrep.2020.02.091 32187560 PMC7218802

[22] Garcia-Flores V. A single-cell atlas of murine reproductive tissues during preterm labor. Cell reports 42 (2023). 10.1016/j.celrep.2022.111846 PMC9946687 36599348

[23] De Micheli A. J., Spector J. A., Elemento O. & Cosgrove B. D. A reference single-cell transcriptomic atlas of human skeletal muscle tissue reveals bifurcated muscle stem cell populations. Skeletal muscle 10, 19 (2020). 32624006 10.1186/s13395-020-00236-3 PMC7336639

[24] Li J. KIR+ CD8+ T cells suppress pathogenic T cells and are active in autoimmune diseases and COVID-19. Science 376, eabi9591 (2022). 10.1126/science.abi9591 PMC8995031 35258337

[25] Burzyn D. A special population of regulatory T cells potentiates muscle repair. Cell 155, 1282–1295 (2013). 24315098 10.1016/j.cell.2013.10.054 PMC3894749

[26] Stadinski B. D. Hydrophobic CDR3 residues promote the development of self-reactive T cells. Nat Immunol 17, 946–955 (2016). 10.1038/ni.3491 27348411 PMC4955740

[27] Harly C., Robert J., Legoux F. & Lantz O. γδ T, NKT, and MAIT Cells During Evolution: Redundancy or Specialized Functions? J Immunol 209, 217–225 (2022). 10.4049/jimmunol.2200105 35821101 PMC7613099

[28] Salou M. A common transcriptomic program acquired in the thymus defines tissue residency of MAIT and NKT subsets. J Exp Med 216, 133–151 (2019). 10.1084/jem.20181483 30518599 PMC6314520

[29] Kuswanto W. Poor repair of skeletal muscle in aging mice reflects a defect in local, interleukin-33-dependent accumulation of regulatory T cells. Immunity 44, 355–367 (2016). 26872699 10.1016/j.immuni.2016.01.009 PMC4764071

[30] DeStefano S. Conserved and tissue-specific immune responses to biologic scaffold implantation. Acta Biomaterialia (2024). 10.1016/j.actbio.2024.06.013 PMC12306723 38879103

[31] Ngo T. B. Intersection of Immunity, Metabolism, and Muscle Regeneration in an Autoimmune-Prone MRL Mouse Model. Advanced Science, 2306961 (2024). 10.1002/advs.202306961 PMC10953568 38192168

[32] Fung-Leung W. P. CD8 is needed for development of cytotoxic T cells but not helper T cells. Cell 65, 443–449 (1991). 10.1016/0092-8674(91)90462-8 1673361

[33] Hildner K. Batf3 deficiency reveals a critical role for CD8α+ dendritic cells in cytotoxic T cell immunity. Science 322, 1097–1100 (2008). 19008445 10.1126/science.1164206 PMC2756611

[34] Anderson M. S. Projection of an immunological self shadow within the thymus by the aire protein. Science 298, 1395–1401 (2002). 12376594 10.1126/science.1075958

[35] Szklarczyk D. The STRING database in 2023: protein–protein association networks and functional enrichment analyses for any sequenced genome of interest. Nucleic acids research 51, D638–D646 (2023). 36370105 10.1093/nar/gkac1000 PMC9825434

