# Supplementary material for: Damage signals preferentially activate killer CD8+/− regulatory T cells to protect injured tissue
Source: bioRxiv. 2025 Jan 14:2025.01.13.632166. Preprint. [Version 1] doi: 10.1101/2025.01.13.632166 (PMC12190476; doi:10.1101/2025.01.13.632166)
Supplement: Supplement 1 [file NIHPP2025.01.13.632166v1-supplement-1.pdf]

bioRxiv preprint doi: https://doi.org/10.1101/2025.01.13.632166; this version posted January 14, 2025. The copyright holder for this preprint (which was not certified by peer review) is the author/funder. This article is a US Government work. It is not subject to copyright under 17 USC 105 and is also made available for use under a CC0 license.

SUPPLEMENTAL FILE S1:

Supplemental Figures S1-S7

Supplemental Table S1-S2

Josyula et al.

bioRxiv preprint doi: https://doi.org/10.1101/2025.01.13.632166; this version posted January 14, 2025. The copyright holder for this preprint (which was not certified by peer review) is the author/funder. This article is a US Government work. It is not subject to copyright under 17 USC 105 and is also made available for use under a CC0 license.

SUPPLEMENTAL FIGURES

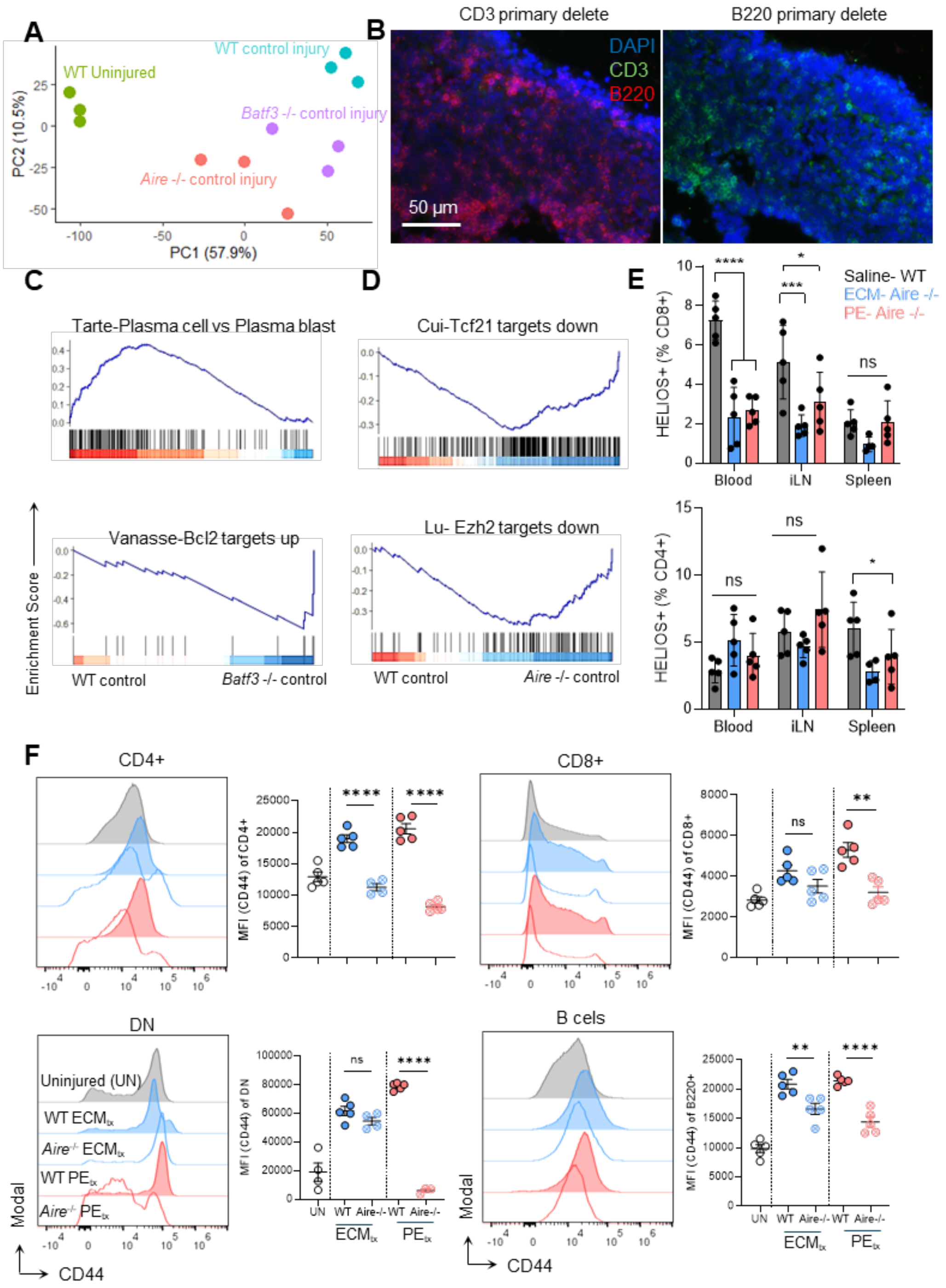

Figure S1 | Transcriptional similarity of *Aire*$^{-/-}$ and *Batf3*$^{-/-}$ mice and HELIOS profile of *Aire*$^{-/-}$ after muscle damage (A) PCA plot of muscle bulk-RNA sequencing data from indicated knockout strains 7 days post injury. (B) Primary delete controls of Immunofluorescent markers identifying B and T cells in muscle. (C-D) GSEA analysis of muscle tissue from *Batf3*$^{-/-}$ (C) and

bioRxiv preprint doi: https://doi.org/10.1101/2025.01.13.632166; this version posted January 14, 2025. The copyright holder for this preprint (which was not certified by peer review) is the author/funder. This article is a US Government work. It is not subject to copyright under 17 USC 105 and is also made available for use under a CC0 license.

Aire$^{-/-}$ (D) mice.(E) HELIOS expression in indicated strains in blood, lymph nodes and spleen at 7 days post-injury. (F) CD44 median fluorescence intensities in indicated lymphocyte populations and treatment groups at 7 days post-injury.

bioRxiv preprint doi: https://doi.org/10.1101/2025.01.13.632166; this version posted January 14, 2025. The copyright holder for this preprint (which was not certified by peer review) is the author/funder. This article is a US Government work. It is not subject to copyright under 17 USC 105 and is also made available for use under a CC0 license.

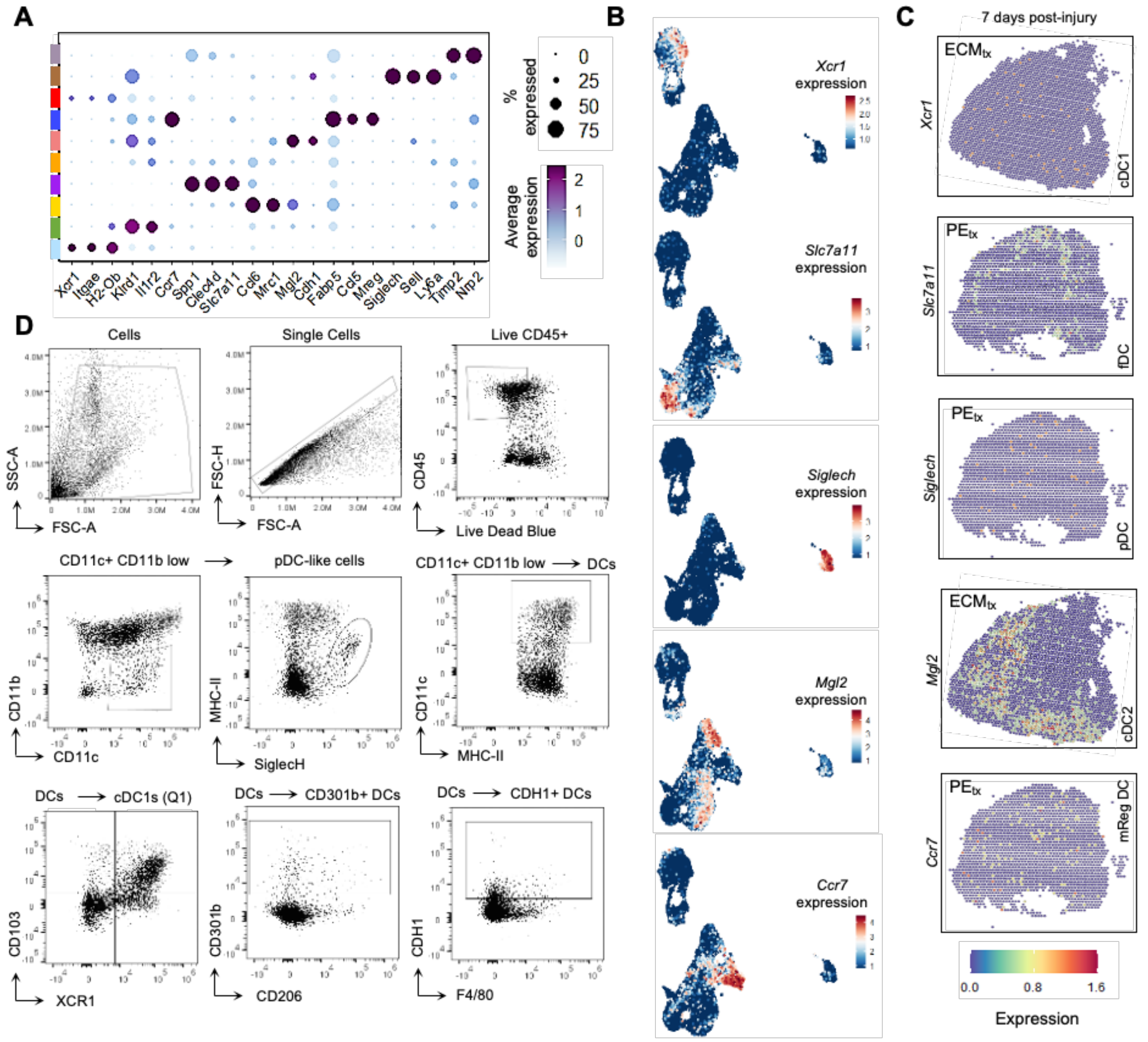

Figure S2 | Single cell transcriptomic cluster identity, spatial distribution of markers and flow cytometry gating scheme of dendritic cell subsets. (A) Dot plot of characteristic dendritic cell markers. (B) Feature plot of characteristic dendritic cell markers. (C) Spatial distribution of characteristic dendritic cell markers. (D) Flow cytometry gating scheme of panel comprising dendritic cell markers.

bioRxiv preprint doi: https://doi.org/10.1101/2025.01.13.632166; this version posted January 14, 2025. The copyright holder for this preprint (which was not certified by peer review) is the author/funder. This article is a US Government work. It is not subject to copyright under 17 USC 105 and is also made available for use under a CC0 license.

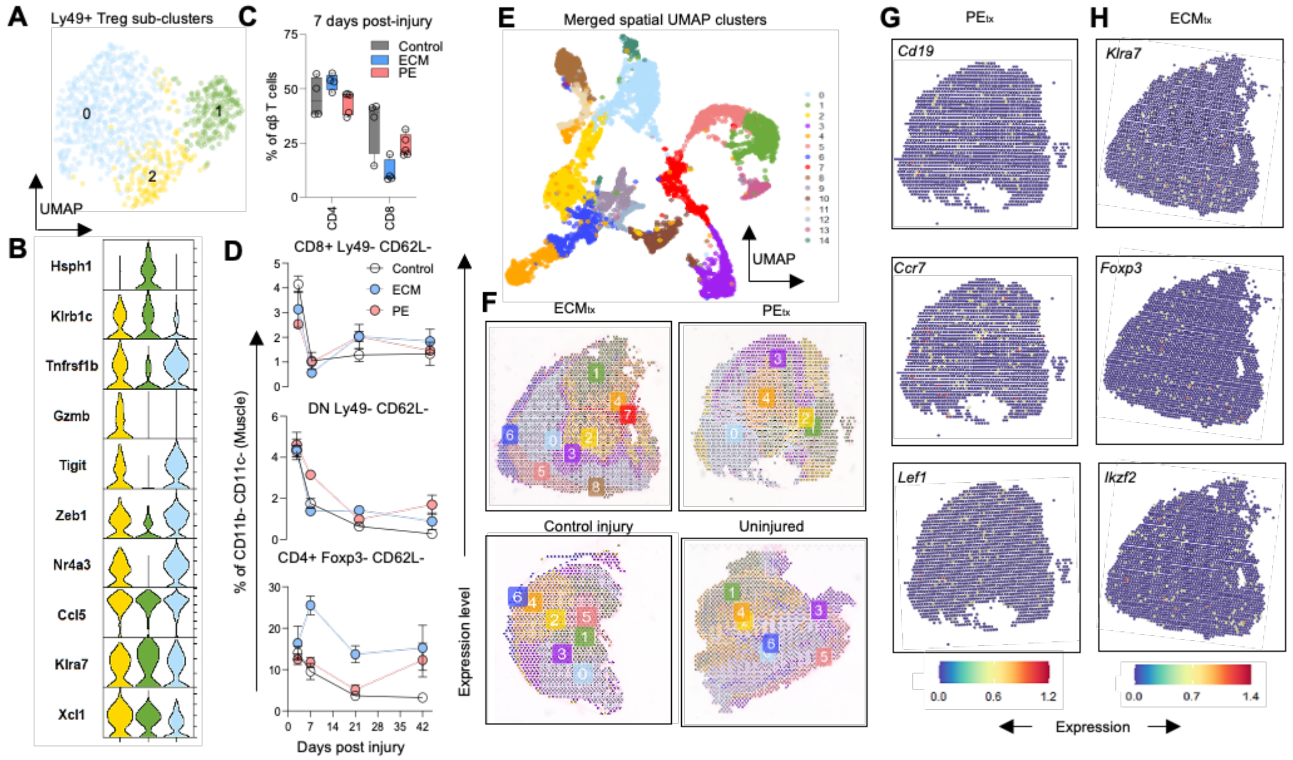

Figure S3 | Ly49+ Treg subclusters, activated T cell accumulation and spatial transcriptomic profiling of T and B cell markers.(A-B) Sub-cluster of Ly49+ T regs (A) and distribution of key markers within sub-clusters (B).(C-D) CD4 and CD8 T cell fractions within muscle tissue 7 days post-injury (C) and accumulation of activated T cells to the muscle (D). (E-F) UMAP clustering (E) and spatial distribution (F) of muscle cells at 7 days post-injury.(G-H) Characteristic markers of enriched cell types in PE_{tx} (G) and ECM_{tx} (H).

bioRxiv preprint doi: https://doi.org/10.1101/2025.01.13.632166; this version posted January 14, 2025. The copyright holder for this preprint (which was not certified by peer review) is the author/funder. This article is a US Government work. It is not subject to copyright under 17 USC 105 and is also made available for use under a CC0 license.

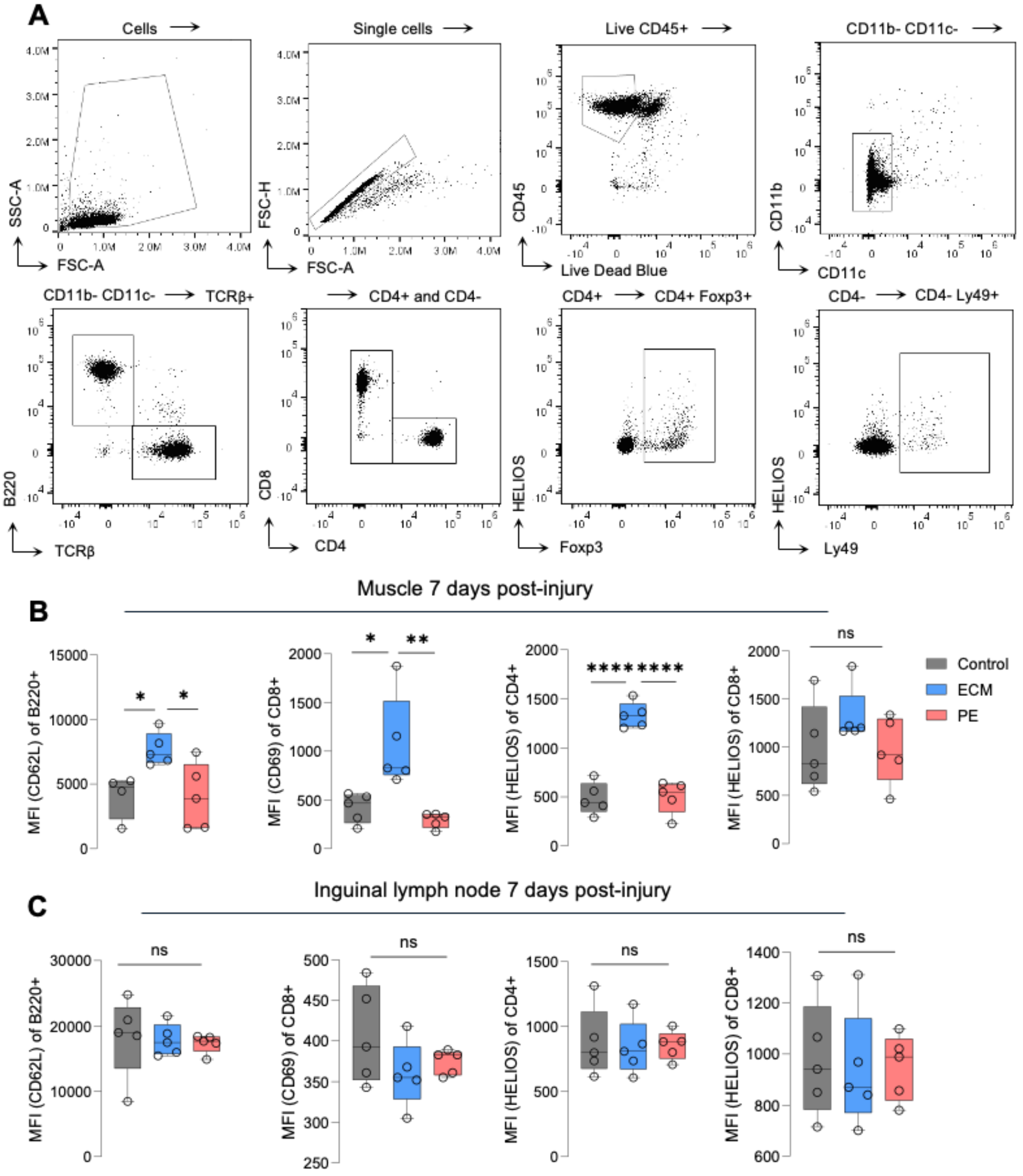

Figure S4 | Flow cytometry gating scheme, activation markers and HELIOS expression profiles of B and T cells. (A) Flow cytometry gating scheme to identify key regulatory T cell subsets and B cells in muscle tissue. (B-C) B cell activation as defined by CD62L shedding, CD69 and HELIOS expression in CD8+ and CD4+ T cells in muscle (B) and lymph nodes (C) at 7 days post-injury.

bioRxiv preprint doi: https://doi.org/10.1101/2025.01.13.632166; this version posted January 14, 2025. The copyright holder for this preprint (which was not certified by peer review) is the author/funder. This article is a US Government work. It is not subject to copyright under 17 USC 105 and is also made available for use under a CC0 license.

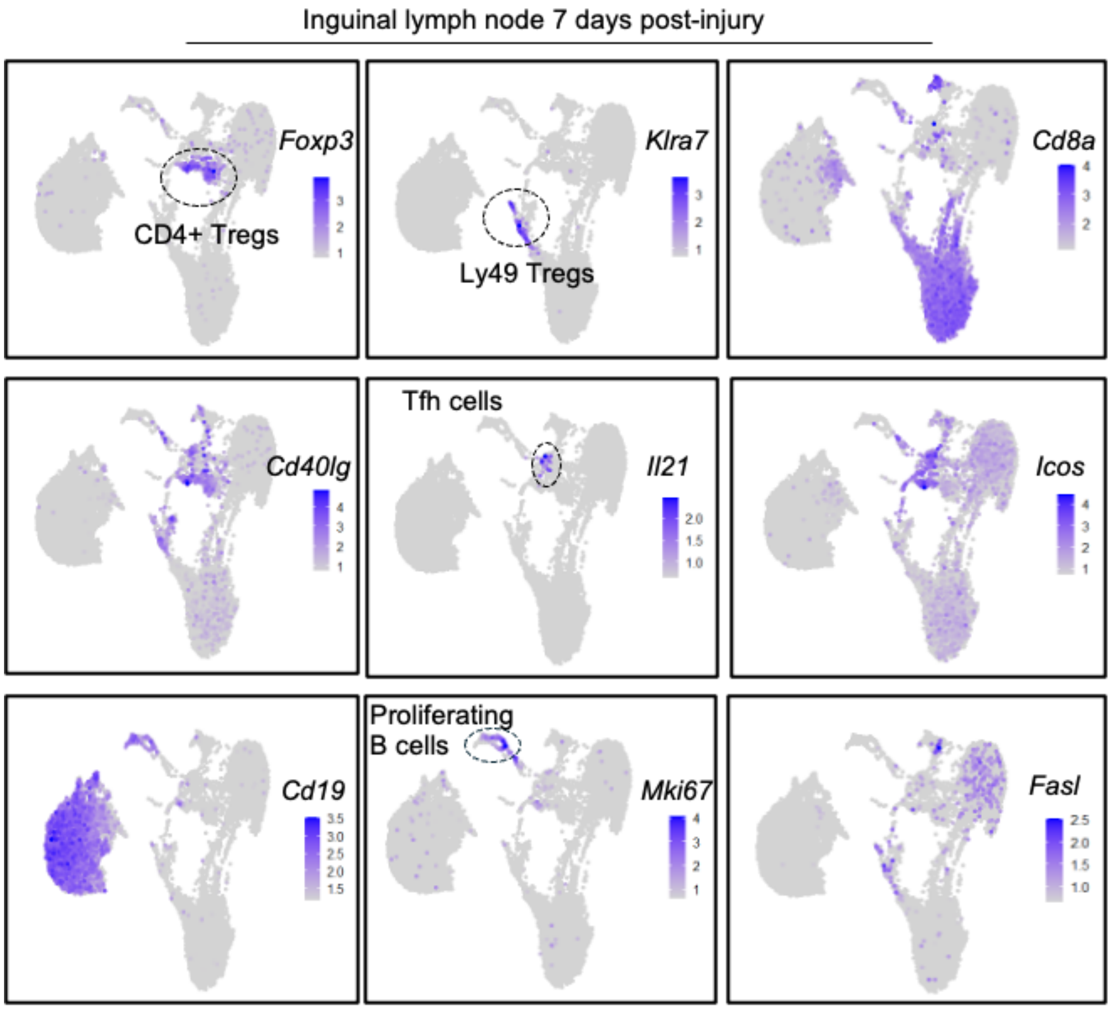

Figure S5 | Inguinal lymph node single cell RNAseq clusters. Uniform manifold approximation projection (UMAP) showing normalized gene expression of key genes in different lymphocyte clusters.

bioRxiv preprint doi: https://doi.org/10.1101/2025.01.13.632166; this version posted January 14, 2025. The copyright holder for this preprint (which was not certified by peer review) is the author/funder. This article is a US Government work. It is not subject to copyright under 17 USC 105 and is also made available for use under a CC0 license.

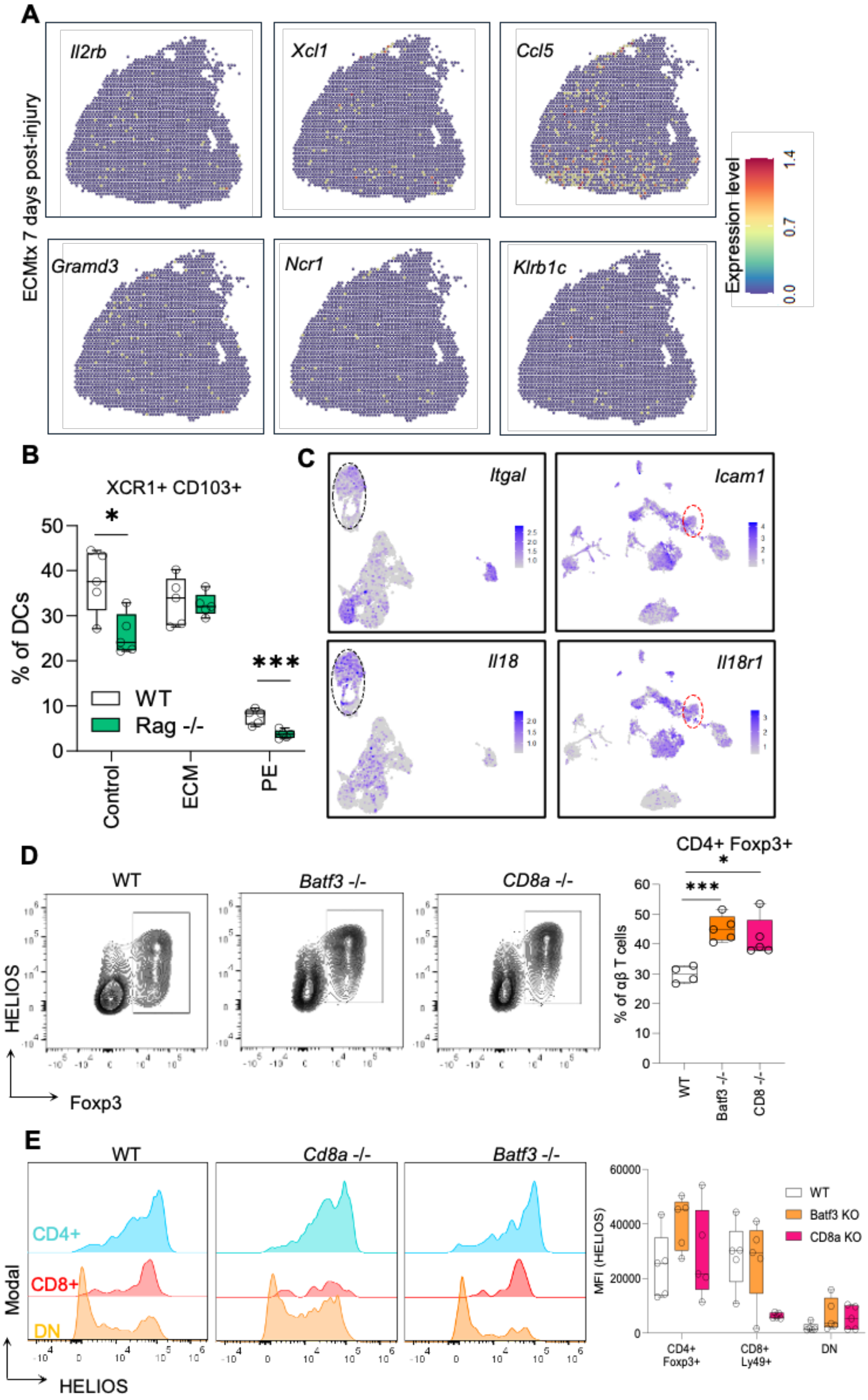

Figure S6 | Spatial distribution, contribution to cDC1 recruitment of Ly49+ killer T regs and HELIOS expression in indicated T cell subsets in *Batf3*$^{-/-}$ and *Cd8a*$^{-/-}$ mice. (A) Spatial

bioRxiv preprint doi: https://doi.org/10.1101/2025.01.13.632166; this version posted January 14, 2025. The copyright holder for this preprint (which was not certified by peer review) is the author/funder. This article is a US Government work. It is not subject to copyright under 17 USC 105 and is also made available for use under a CC0 license.

distribution of select Ly49+ Treg markers. (B) Muscle recruitment of XCR1+ CD103+ in WT and *Rag*$^{-/-}$ mice across treatment groups. (C) Complimentary expression of receptor-ligand pairs in cDC1 and Ly49+ Treg cells. (D) Muscle recruitment of CD4+ Foxp3+ Tregs in WT, *Batf3* $^{-/-}$ and *Cd8a*$^{-/-}$ mice. (E) HELIOS expression in WT, *Batf3* $^{-/-}$ and *Cd8a*$^{-/-}$ mice in the muscle across indicated T cell subsets.

bioRxiv preprint doi: https://doi.org/10.1101/2025.01.13.632166; this version posted January 14, 2025. The copyright holder for this preprint (which was not certified by peer review) is the author/funder. This article is a US Government work. It is not subject to copyright under 17 USC 105 and is also made available for use under a CC0 license.

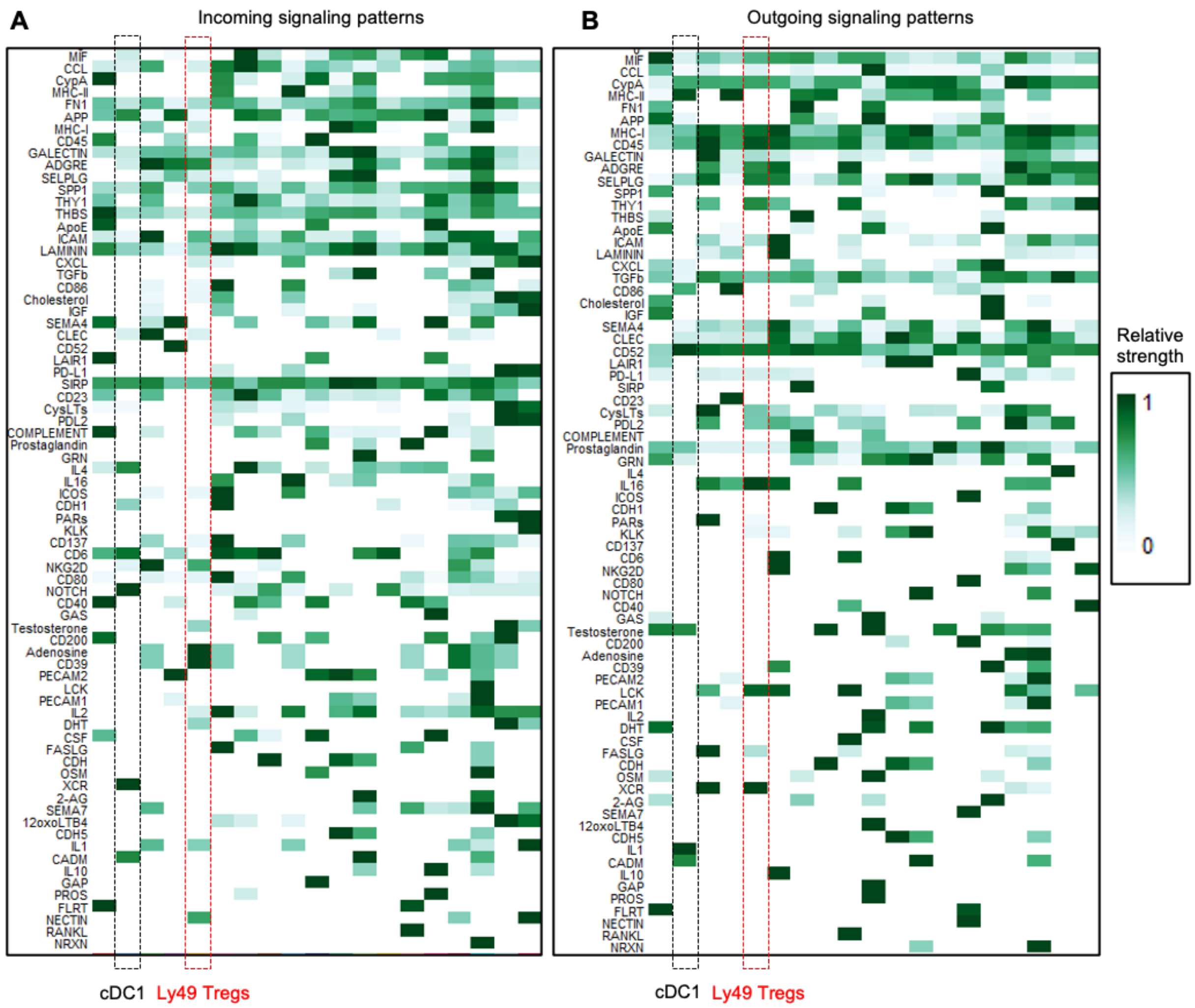

Figure S7 | CellChat Analysis of scRNAseq datasets. (A) Incoming and (B) Outgoing signaling patterns of key antigen presenting cell and lymphocyte clusters.

bioRxiv preprint doi: https://doi.org/10.1101/2025.01.13.632166; this version posted January 14, 2025. The copyright holder for this preprint (which was not certified by peer review) is the author/funder. This article is a US Government work. It is not subject to copyright under 17 USC 105 and is also made available for use under a CC0 license.

**Cell type**	**Signature genes**
Conventional dendritic cells type 1 (cDC1)	*Xcr1, Itgae, Clec9a, Irf8, Batf3, Ckb, Cebp3, Capg3*
$CD8^{+/-}Ly49^{+}$ regulatory T cells ($CD8^{+/-}Ly49^{+}$ Tregs)	*Xcl1, Ccl5, Ikzf2, Cd3e, Klra7, Klra4, Klra6, Serpinb9, Bach2, Ctla2a, Nkg7, Ly6c2, Il2rb, Fasl, Ctsw, Klra1, Cd7, Cd27, Gramd3*
$CD4^{+}FoxP3^{+}$ regulatory T cells ($CD4^{+}$ Tregs)	*Cd4, Foxp3, Ikzf2, Il2rb, Areg, Icos, Gzmb, Tigit, Ctla4, Tnfrsf9, Klrg1, Ccr8*

Table S1 | Signatures genes corresponding to key DC and T cell clusters in single cell RNA sequencing and spatial transcriptomics data.

bioRxiv preprint doi: https://doi.org/10.1101/2025.01.13.632166; this version posted January 14, 2025. The copyright holder for this preprint (which was not certified by peer review) is the author/funder. This article is a US Government work. It is not subject to copyright under 17 USC 105 and is also made available for use under a CC0 license.

Marker	Fluorophore	Clone	Source
CD45	BUV395	30-F11	BD Biosciences
CD11b	BV510	M1/70	BD Biosciences
CD11c	PE-Cy5	N418	BD Biosciences
TCRb	BUV563	H57-597	BD Biosciences
CD4	APC	GK1.5	BD Biosciences
CD8	AF532	53-6.7	Invitrogen
CD62L	APC-Cy7	MEL-14	BD Biosciences
Ly49	PE	14B11	BioLegend
MHC II	BUV496	2G9	BD Biosciences
CD69	BV711	H1.2F3	BioLegend
B220	APC Fire810	RA3-6B2	BioLegend
Foxp3	AF488	150D	BioLegend
HELIOS	PE-Cy7	22F6	BioLegend
Viability	Live-Dead Blue		BD Biosciences

Marker	Fluorophore	Clone	Source
CD45	AF488	I3/2.3	BD Biosciences
CD11c	PE	N418	BD Biosciences
CD11b	BV510	M1/70 and HL3	BD Biosciences
MHC II	BV421	M5/114.15.2	BD Biosciences
F4/80	APC-Cy7	BM8	BD Biosciences
Siglec F	BV 786	E50-2440	BD Biosciences
Viability	7-AAD		BD Biosciences

Marker	Fluorophore	Clone	Source
CD45	BUV395	30-F11	BD Biosciences
CD11b	BUV661	M1/70	BD Biosciences
CD11c	PE-Cy5	N418	BD Biosciences
F480	BUV805	T45-2342	BD Biosciences
SiglecH	AF647	551	BD Biosciences
XCR1	BV650	ZET	BioLegend
CD103	APC-R700	M290	BD Biosciences
CD301b	RB780	URA-1	BD Biosciences
Cdh1	PE-Dazzle 594	DECMA-1	BioLegend
CD206	BV421	MMR	BioLegend
MHC II	BUV496	2G9	BD Biosciences
SiglecF	BV605	E50-2440	BD Biosciences
B220	APC Fire810	RA3-6B2	BioLegend
Viability	Live-Dead Blue		BD Biosciences

Marker	Fluorophore	Clone	Source
CD45	AF488	I3/2.3	BD Biosciences
TCRb	PE-Cy7	H57-597	BD Biosciences
B220	BV421	RA3-6B2	BD Biosciences
CD11b	BV510	M1/70	BD Biosciences
NK1.1	BV786	PK136	BD Biosciences
CD62L	APC-Cy7	MEL-14	BD Biosciences
TCRgd	PE	GL-3	BD Biosciences
CD11c	BV510	HL3	BD Biosciences
Viability	7-AAD		BD Biosciences

Supplemental Table S2 | Antibody panels used in flow cytometry analysis and sorting
